# Synthetic Control of Metabolic States in Pseudomonas putida by Tuning Polyhydroxyalkanoate Cycle

**DOI:** 10.1128/mbio.01794-21

**Published:** 2022-01-18

**Authors:** Maria-Tsampika Manoli, Juan Nogales, Auxiliadora Prieto

**Affiliations:** a Interdisciplinary Platform for Sustainable Plastics Towards a Circular Economy–Spanish National Research Council (SusPlast‐CSIC), Madrid, Spain; b Polymer Biotechnology Group, Microbial and Plant Biotechnology Department, Biological Research Centre Margarita Salas, CIB-CSIC, Madrid, Spain; c Systems Biotechnology Group (SBG), Department of Systems Biology, National Centre for Biotechnology (CNB-CSIC), Madrid, Spain; University of California, Berkeley

**Keywords:** metabolic robustness, *Pseudomonas*, polyhydroxyalkanoates, PHA metabolism, PHA depolymerase, oxidative stress, flux balance analysis

## Abstract

Polyhydroxyalkanoates (PHAs) are polyesters produced by numerous microorganisms for energy and carbon storage. Simultaneous synthesis and degradation of PHA drives a dynamic cycle linked to the central carbon metabolism, which modulates numerous and diverse bacterial processes, such as stress endurance, pathogenesis, and persistence. Here, we analyze the role of the PHA cycle in conferring robustness to the model bacterium P. putida KT2440. To assess the effect of this cycle in the cell, we began by constructing a PHA depolymerase (PhaZ) mutant strain that had its PHA cycle blocked. We then restored the flux through the cycle in the context of an engineered library of P. putida strains harboring differential levels of PhaZ. High-throughput phenotyping analyses of this collection of strains revealed significant changes in response to PHA cycle performance impacting cell number and size, PHA accumulation, and production of extracellular (*R*)-hydroxyalkanoic acids. To understand the metabolic changes at the system level due to PHA turnover, we contextualized these physiological data using the genome-scale metabolic model *i*JN1411. Model-based predictions suggest successive metabolic steady states during the growth curve and an important carbon flux rerouting driven by the activity of the PHA cycle. Overall, we demonstrate that modulating the activity of the PHA cycle gives us control over the carbon metabolism of P. putida, which in turn will give us the ability to tailor cellular mechanisms driving stress tolerance, e.g., defenses against oxidative stress, and any potential biotechnological applications.

## INTRODUCTION

The group Pseudomonas comprises a heterogeneous and large number (>100) of Gram-negative, aerobic gammaproteobacterial species (1). Pseudomonas has a robust metabolism and is physiologically versatile, which enables fast adaptation to fluctuating environments and evolvability while facilitating colonization of diverse niches, many of them often hostile to other bacterial genera (2). Most of these relevant traits of pseudomonads are powered by their primary and accessory metabolisms and are in their core genome, which comprises around 1,000 genes (3–7). Boosted by these exceptional metabolic features, Pseudomonas spp. have emerged as a notable bacterial group, sparking growing interest in fields as diverse as plant and human diseases (8, 9), agriculture, biodegradation, and industrial biotechnology (5, 10, 11). Despite said interest and the intense scrutiny of Pseudomonas’ metabolism in recent years (4, 12), the molecular basis underpinning dynamic control of their physiology under disturbance conditions remains largely unknown.

Among this diverse group, Pseudomonas putida is a paradigm of the “cosmopolitan bacterium” that is well-known for its robust metabolism and stress resilience (13–15). P. putida is often isolated in polluted soil and aquatic environments, a fact that has driven extensive research into its stress tolerance mechanisms and adaptability. Strain KT2440 is considered a microbial biocatalyst and has been used in multiple metabolic engineering endeavors, supported by rational genetic modifications using an ever-increasing number of genetic tools and genome-scale models (GEMs) (12, 16–18). Among other industrial applications, KT2440 is a paradigmatic model for production of bioproducts such as bacterial polyesters or polyhydroxyalkanoates (PHAs) (10, 19, 20).

**FIG 1:**
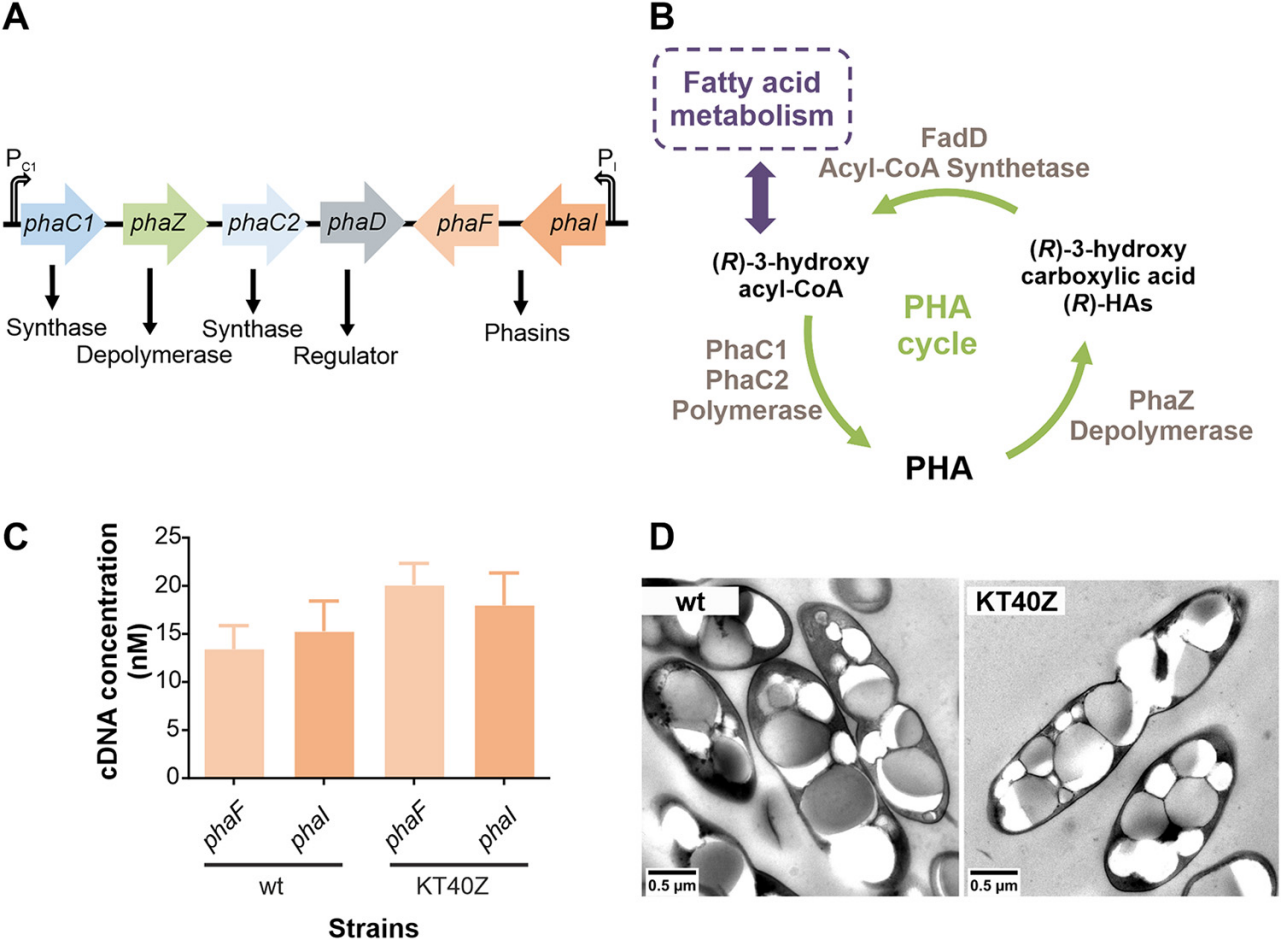
Construction and initial phenotyping of *phaZ* null depolymerase mutant. (A) *pha* gene cluster in P. putida KT2440. Both operons, *phaC1ZC2D* and *phaIF*, are transcripted divergently (31). The transcription of these genes is driven by global and effector-specific regulators (reviewed in reference 20). (B) PHA cycle in P. putida, where the key players are the PHA polymerases (PhaC1 and PhaC2), PHA depolymerase (PhaZ), and acyl-CoA synthetase (FadD). (*R*)-3-hydroxyacyl-CoA is the substrate for PHA polymerases and for the enzymes responsible for the metabolism of fatty acids. (C) Quantification of phasin transcription levels by qRT-PCR experiments (*phaF*, shaded light pink bars, and *phaI*, shaded dark pink bars) for the wild type (wt) and KT40Z. Strains were monitored after 6 h of growth under PHA accumulation conditions. One-way ANOVA was performed, and no significant differences on the phasin transcription levels were observed between the two strains. (D) Transmission electronic microscopy (TEM) pictures of wild-type (wt; KT2440) and KT40Z strains after 24 h of growth under PHA accumulation conditions are shown. The scale bar is 0.5 μm.

PHAs are accumulated as reserve storage granules in the cell cytoplasm, mainly under nutrient imbalances like carbon excess coupled to limited availability of nutrients such as nitrogen and phosphorous, among others (21, 22). Granules are coated by the granule-associated proteins (GAPs) involved in PHA metabolism and regulation, i.e., polymerases, depolymerases, phasins, and others with similar functions (23–26). In P. putida, some of these GAPs and the PhaD transcriptional activator are coded in a *pha* gene cluster, which is well conserved throughout the mcl-PHA (medium-chain-length) producer strains (Fig. 1) (26). The bacterial PHA metabolic machinery is closely connected to central and peripheral metabolic pathways supplying (*R*)-3-hydroxyacyl-coenzyme A [(*R*)-HA-CoA] as a substrate for PHA polymerases. In the case of pseudomonads, PHA metabolism relies on the β-oxidation pathway and *de novo* fatty acid biosynthesis for the conversion of fatty acid and non-fatty acid precursors into different (*R*)-HA-CoAs for mcl-PHA biosynthesis. PHA metabolism is controlled via a multilevel regulatory network driven by global regulators linked to central carbon metabolism and *pha*-specific regulators in the *pha* cluster (Fig. 1A and recently reviewed in reference 20).

Key GAPs mediating the PHA cycle in P. putida are the PHA polymerases (PhaC1 and PhaC2) and the PHA depolymerase (PhaZ). The former synthesize and the latter degrades PHA by releasing 3-hydroxyalkanoic acids [(*R*)-HAs or free monomers]. Acyl-CoA synthetase (FadD1) subsequently reactivates the free monomers into (*R*)-HA-CoA in an ATP-dependent reaction (20, 26) (Fig. 1B). This process implies PHA turnover, where both synthesis and degradation of the polymer are active simultaneously (19, 23). Consequently, it has been suggested that, in addition to its primary carbon storage function, this bidirectional flux could provide a certain buffering capability, granting the PHA cycle the ability to control carbon and energy spillage in P. putida (22). Along these lines, it has also been suggested that the PHA cycle acts as a homeostatic cycle, providing stability and metabolic fitness under environmental perturbations. In other words, the PHA cycle might be a metabolic capacitor connecting catabolism and anabolism with P. putida’s central metabolism. Such cycles have been defined as robustness cycles (27–30).

The production of PHA is a metabolic feature largely present in Pseudomonas, highlighting the role of PHA metabolism in the evolutionary success of this important bacterial genus (6).

In an attempt to find metabolic features providing dynamic control over metabolism in Pseudomonas, in this work we assess the role of the PHA cycle as a robustness cycle in P. putida KT2440. This required a multidisciplinary approach involving systems and synthetic biology to tune the flux of carbon through the PHA cycle by adjusting the flux through the PhaZ reaction. We also provide solid evidence that the PHA cycle plays a key metabolic role in the induction of carbon-flux rerouting under perturbations such as oxidative stress.

## RESULTS

### Engineering a synthetic tuning of PHA turnover.

PHA turnover functionality ensures that the dynamic flux of (*R*)-HAs toward the central metabolism is available when needed. To study the effect of a defective PHA cycle on the physiology of P. putida, we started by constructing a host strain that was missing the PhaZ depolymerase-encoding gene and therefore was unable to hydrolyze PHA. Since the *pha* cluster is arranged in two convergent operons (*phaC1ZC2D* and *phaFI*), deleting the *phaZ* gene might trigger polar effects on the transcription level of the whole cluster due to the defective expression of the transcriptional activator, *phaD*, that controls the activity of promoters P_C1_ and P_I_ (31) (Fig. 1A). Therefore, the scar sequence of the *phaZ* deletion mutant (KT40Z) strain was carefully designed and verified via sequencing to ensure the in-frame expression of the remaining *pha* genes (see Fig. S1 in the supplemental material). The absence of polar effects was further verified by quantitative reverse transcription-PCR (qRT-PCR) experiments, monitoring the transcription levels of *phaF* and *phaI* genes in KT40Z and KT2440 cells growing under PHA accumulation conditions (mid-exponential phase) (Fig. 1C). No major differences were observed between the strains; hence, the innocuous genotype of the KT40Z strain was validated. The growth profile of KT40Z (see below) was similar to that of the wild-type strain. However, the *phaZ* mutant strain did not release (*R*)-HAs (Table 1), confirming the generation of an interrupted PHA cycle. We then analyzed the phenotype of the knockout strain in terms of PHA accumulation and granule cell localization using transmission electronic microscopy. As expected, no effect on PHA production was observed, and the KT40Z cells were able to accumulate PHA as efficiently as the wild type after 24 h of growth (Fig. 1D).

**TABLE 1:** Physiological data after 24 h of growth under PHA accumulation conditions^*a*^

Strain	Total biomass (g/L)	PHA		Residual biomass (g/L)	(*R*)-HA concn (g/L)	No. of viable cells (10^8^/ml)	Growth rate (h^−1^)
		% CDW	Concn (g/L)				
KT2440	1.3 ± 0.1	71.7 ± 3.1	1.0 ± 0.1	0.4 ± 0.0	0.2 ± 0.0	1.8 ± 0.6	0.31 ± 0.02
KT40Z	1.4 ± 0.0	72.3 ± 3.3	1.0 ± 0.1	0.4 ± 0.0	0.0 ± 0.0	1.2 ± 0.5	0.31 ± 0.01
KT2440 Δ*pha*	0.6 ± 0.1	0.0 ± 0.0	<0.01	0.6 ± 0.1	0.0 ± 0.0	22.3 ± 4.8	0.34 ± 0.00
M1	1.3 ± 0.1	70.2 ± 3.7	0.9 ± 0.1	0.4 ± 0.0	0.3 ± 0.0	4.1 ± 0.3	0.30 ± 0.01
M2	0.6 ± 0.0	0.3 ± 0.4	<0.01	0.6 ± 0.0	0.5 ± 0.1	16.5 ± 3.1	0.29 ± 0.00
M3	0.7 ± 0.1	0.4 ± 0.4	<0.01	0.7 ± 0.1	0.6 ± 0.0	16.8 ± 3.1	0.31 ± 0.00
M4	0.5 ± 0.0	0.7 ± 1.0	<0.01	0.5 ± 0.0	0.5 ± 0.0	15.4 ± 3.6	0.35 ± 0.02

a Sodium octanoate (mM) was not detected in the culture supernatant of any of the strains tested.

10.1128/mBio.01794-21.5 FIG S1 Schematic representation of the construction of the *phaZ* deletion mutant. Oligonucleotides used for the deletion process are shown with black arrows and detailed in Table S2. During deletion, the Z1-phaZ-For and Z1-phaZ-Rev primers were used to obtain the Z1 upstream homology region, and Z3-phaZ-For and Z3-phaZ-Rev primers were used to obtain the Z3 downstream homology region. For the second recombination process verification, the external phaC1-For and phaC2-Rev primers were used. The postdeletion remaining nucleotide sequence is shown. The stop codons are highlighted in boldface blue type and the start codons in boldface underlined. The first stop codon is contained in the *phaC1* gene and the second in the *phaZ* gene. The first start codon is contained in *phaZ* and the second in *phaC2*. The ribosome binding site (RBS) is shown in red. The *phaZ* remaining nucleotide sequence is shown in orange. Download FIG S1, TIF file, 2.4 MB. Copyright © 2022 Manoli et al. 2022 Manoli et al. https://creativecommons.org/licenses/by/4.0/ This content is distributed under the terms of the Creative Commons Attribution 4.0 International license.

10.1128/mBio.01794-21.2 TABLE S2 Oligonucleotides used in this study. The site for restriction enzymes is underlined. The following restriction enzymes were used: AvrII (CCTAGG), BamHI-HF (GGATCC), HindIII-HF (AAGCTT), and XbaI (TCTAGA). Download Table S2, DOCX file, 0.04 MB. Copyright © 2022 Manoli et al. 2022 Manoli et al. https://creativecommons.org/licenses/by/4.0/ This content is distributed under the terms of the Creative Commons Attribution 4.0 International license.

**FIG 2:**
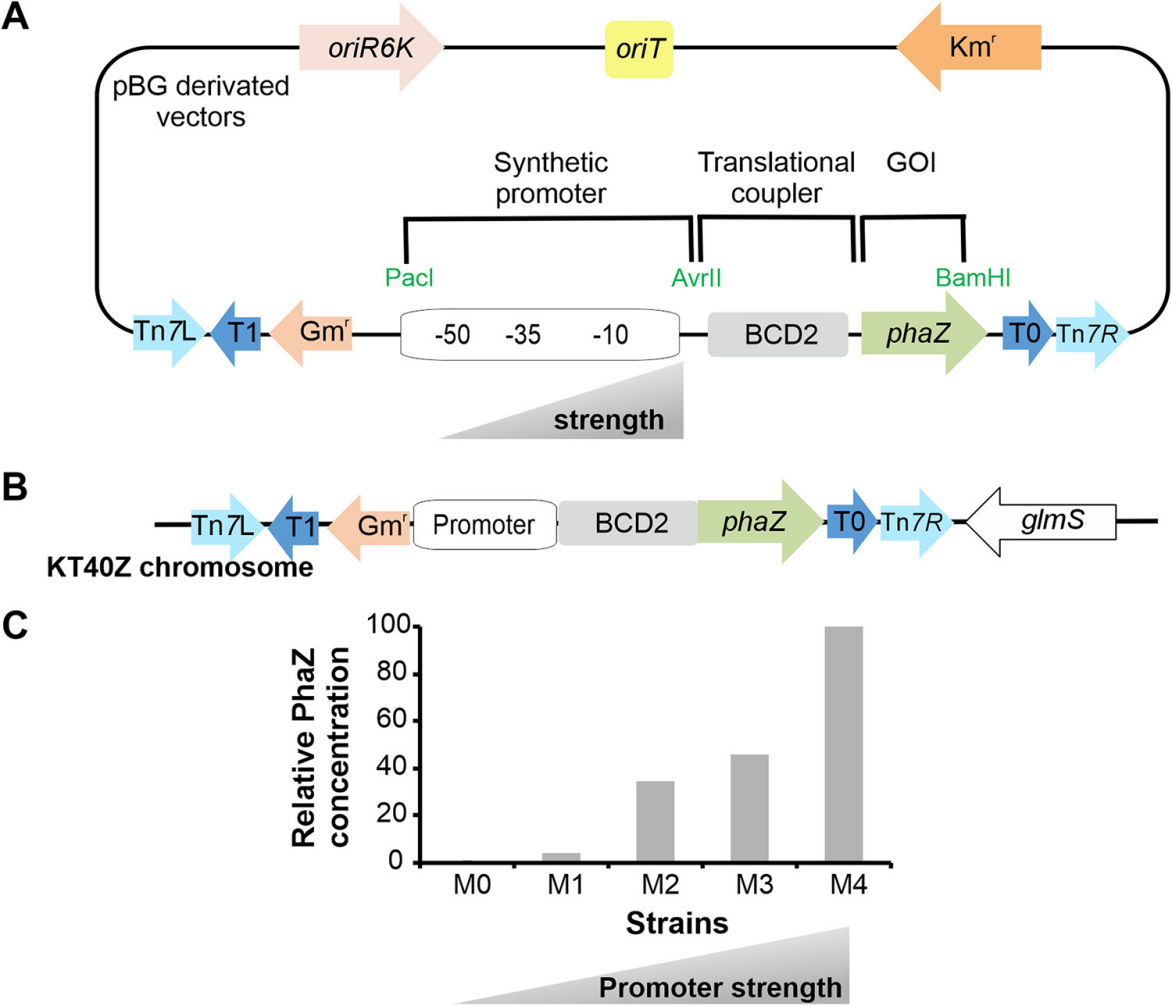
Construction and validation of a library of strains driving differential PhaZ production levels. (A) Structural organization of the pBG derivative plasmids, including their origin of replication (oriR6K; light pink), the origin of transfer (oriT; yellow), the kanamycin-resistant marker (Km^r^; orange), a Tn*7* module with two transposase recognition sites (Tn*7*L, Tn*7*R; light blue), a module bearing two terminators (T1, T0; dark blue), and a gentamicin-resistant marker (Gm^r^; pink). The cargo includes three modules, i.e., the synthetic promoter, the translational coupler (BCD2; gray), and *phaZ* (green). Key restriction enzymes flanking the functional modules are also indicated (green letters). (B) Schematic representation of the chromosomal integration of pBG-derivative vectors in KT40Z background. (C) Relative PhaZ production levels in culture are indicated and measured by Western blotting (Fig. S2). The library of strains named M0 to M4, from lowest (M0) to highest (M4) promoter activity. Strain KT40Z was used as a negative control. The signal intensities of PhaZ production levels were quantified using Image J software. For the calculations, the OD_600_ equivalent load in each case was taken into account, and the data were normalized to M4 production levels (Fig. S2).

To finely tune the flux through the PHA cycle, we set up a library of P. putida strains harboring differential PhaZ production levels (Fig. 2). We began constructing a collection of plasmids where the only variable was the strength of the constitutive promoters driving the expression of the *phaZ* gene (Fig. 2A and Table S1). The collection of vectors was then specifically integrated in the genome of P. putida KT40Z using mini-Tn*7* transposons, resulting in a library of P. putida strains with expected differential expression of *phaZ* (Fig. 2B). The strains were named M0 to M4, where M0 displayed the lowest and M4 the highest promoter strength. Finally, the library of P. putida strains was validated using Western blot analysis to monitor the production of PhaZ from whole-cell extracts recovered during mid-exponential growth (OD_600_ of 0.6) (Fig. S2).

10.1128/mBio.01794-21.1 TABLE S1 Strains and plasmids used in this study. A point mutation (A→G) was observed in the promoter sequence leading to 14d: TTAATTAATCTACTTGACATCCGACATTCGCGACTGTATAATAAGTTG**G**CCTAGG. Download Table S1, DOCX file, 0.05 MB. Copyright © 2022 Manoli et al. 2022 Manoli et al. https://creativecommons.org/licenses/by/4.0/ This content is distributed under the terms of the Creative Commons Attribution 4.0 International license.

10.1128/mBio.01794-21.6 FIG S2 Anti-PhaZ Western blots from whole-cell extracts collected in mid-exponential phase (OD_600_, 0.6). The OD_600_ equivalent load in each case is annotated. The library of strains is named M0 to M4, with promoter activity ranging between M0 (lowest) and M4 (highest). Strain KT40Z was used as a negative control. Download FIG S2, TIF file, 2.1 MB. Copyright © 2022 Manoli et al. 2022 Manoli et al. https://creativecommons.org/licenses/by/4.0/ This content is distributed under the terms of the Creative Commons Attribution 4.0 International license.

As expected, a positive correlation was found between the promoter strength and the PhaZ levels, where M0 and M4 exhibited the lowest and the highest promoter activity and PhaZ levels, respectively. PhaZ production could not be detected in the M0 strain, while M1 to M4 strains successfully produced increasing levels of PhaZ (Fig. 2C). On the other hand, PhaZ was not detected in cell extracts from the wild-type strain (data not shown), which confirmed the low transcription rate of the *phaZ* gene under these growth conditions (32).

### Increasing the flux through the PHA cycle leads to significant physiological and phenotypical changes in P. putida.

**FIG 3:**
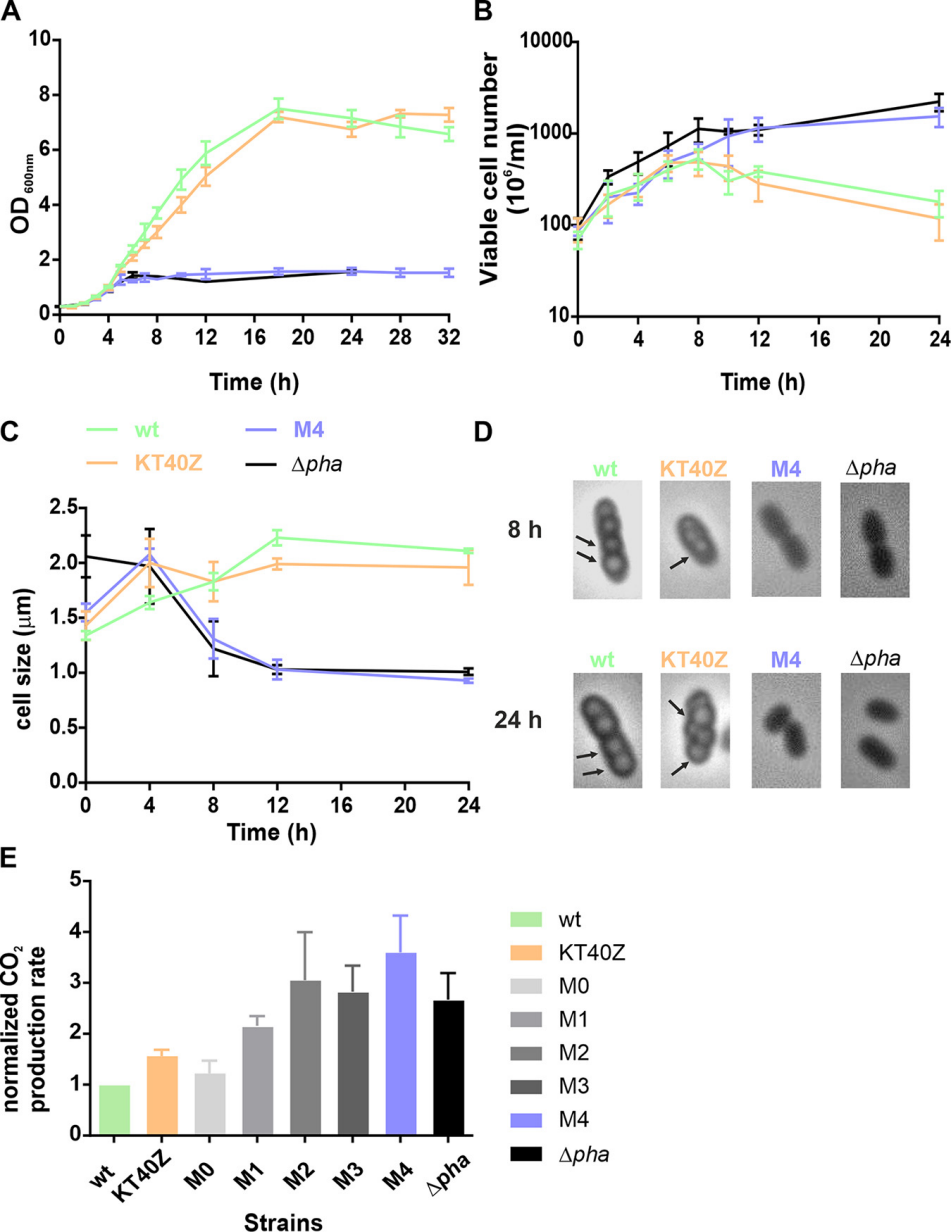
Growth characteristics under PHA accumulation conditions. (A) Growth curves. (B) Viable cell number over time. (C) Cell size quantification over time. (D) Optical microscopy pictures after 8 and 24 h of growth. (E) CO_2_ production rate normalized to wild-type values. The strains tested were KT2440 (green), KT40Z (orange), M4 (blue), KT2440 Δ*pha* (black), and M0 to M3 (shaded gray). The scale bar of the pictures is 2 μm. PHA granules are indicated with arrows.

To investigate the impact of differential PhaZ production levels on the metabolism of P. putida, we next carried out a battery of high-throughput phenotypic analyses that involved growing M0 to M4 strains under optimal PHA accumulation conditions (see Materials and Methods) (19). Key growth parameters were monitored along the growth curve using the wild-type strain and a PHA-defective strain with the *pha* cluster entirely deleted (KT2440 Δ*pha*) as controls (Fig. 3 and Table 1). We observed large differences regarding PHA production properties and growth performance among the different strains. According to these observations, the strains could be classified into two main categories, (i) strains that were able to accumulate PHA (KT2440, KT40Z, M0, and M1) and (ii) strains lacking this ability (M2, M3, M4, and KT2440 Δ*pha*). While PHA-accumulating strains reached 70 to 72% PHA cell dry weight (CDW) after 24 h of growth, P. putida strains harboring high constitutive PhaZ production did not accumulate PHA and instead released higher concentrations of (*R*)-HAs due to a higher PHA depolymerization rate (described below). As expected, the control KT2440 Δ*pha* strain resulted in no PHA accumulation and no (*R*)-HAs were released, since the whole *pha* machinery, including the PhaC polymerase, was missing (Fig. 3 and Table 1). Monitoring total biomass in these experiments returned mixed information, because the PHA produced (grams per liter) adds to the cell biomass (free of PHA), which is referred to as residual biomass. Hence, the higher PHA production capabilities of strains KT2440, KT40Z, M0, and M1 translate into greater total biomass compared to strains M2 to M4. Residual biomass reached concentrations between 0.4 and 0.7 g/L, with some differences found among the strains (Table 1).

Overall, M2 to M4 and KT2440 Δ*pha* strains displayed a higher proportion of residual biomass coupled to a lack of PHA accumulation due to increased depolymerization in the former and absence of the *pha* machinery in the latter. Interestingly, these strains’ inability to accumulate PHA led to a log increase in the number of viable cells after 24 h of growth compared to the strains that were able to accumulate PHA (e.g., KT2440 and KT40Z) (Table 1). In strains M2 to M4, the trend was toward a decrease in optical density at 600 nm (OD_600_) and smaller cell size. In fact, strains M2 to M4 were half the size of the wild-type strain after 8 h of growth (Fig. 3). The decrease in optical density is explained by the absence of PHA accumulation, since PHA producers generally display an opaque phenotype. In this sense, since PHA content disturbs cell’s optical density, the evolution of residual biomass was used for the calculation of growth rates, which turned out to be similar among the strains (Table 1).

To fully understand how the carbon cycle functions in our strains, we monitored levels of residual octanoate and secreted (*R*)-HAs in the supernatants all along the growth curves. At time zero, residual biomass for all strains was between 0.08 and 0.09 g/L, with no PHA or (*R*)-HAs observed either in the culture pellet or the supernatant. No significant amounts of octanoate were detected after 24 h of growth, which suggests that the carbon source was depleted in all cases. Regarding (*R*)-HAs released as a consequence of PhaZ activity, M2 to M4 strains produced up to 0.6 g/L of (*R*)-HAs compared to 0.2 g/L in the wild-type strain after 24 h of growth (Table 1). As anticipated, no (*R*)-HA production was observed in the absence of *phaZ*.

Overall, the smaller total biomass observed with strains M2 to M4, even when accounting for the carbon transformed into (*R*)-HAs, indicates a loss of carbon in the form of extracellular metabolite accumulation [other than (*R*)-HAs] and/or as increased CO_2_ production. To fill this gap, the culture supernatant of all strains after 24 h of growth was analyzed using high-performance liquid chromatography (HPLC) (see Materials and Methods). No significant amounts of any of the metabolites tested were detected (e.g., acetate, pyruvate, succinate, etc.). However, respirometry experiments targeting *in vivo* determination of CO_2_ revealed increased CO_2_ production levels in strains M2 to M4 compared to the wild type after 24 h of growth. In the absence of PHA accumulation, these strains reached 2.8 to 3.6 times higher CO_2_ production than the wild type (Fig. 3E), which strongly suggested a carbon loss due to increased PhaZ flux.

### Construction and validation of condition-specific metabolic models.

**FIG 4:**
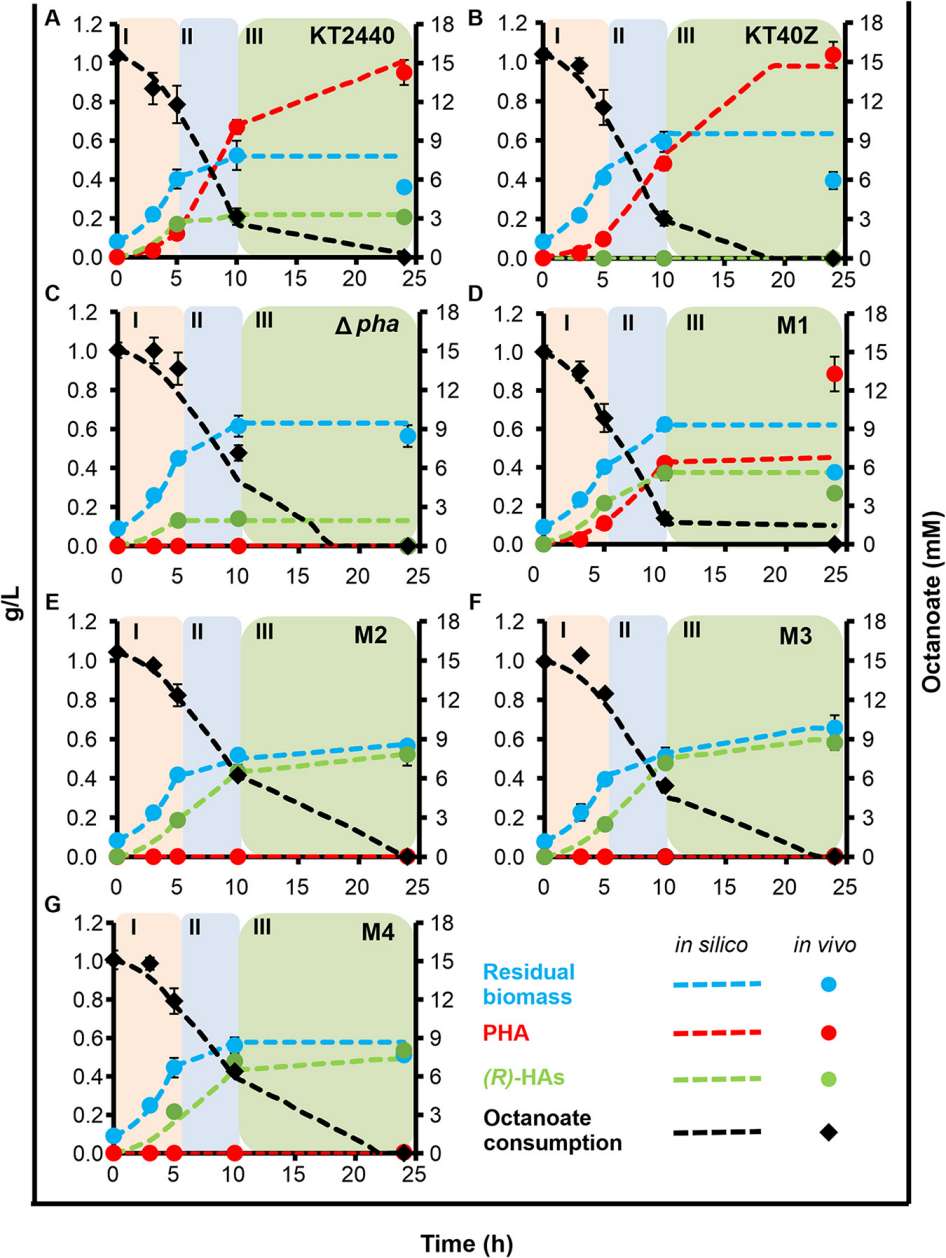
Condition-specific model validation for KT2440, KT40Z, KT2440 Δ*pha*, and M1 to M4 strains using flux balance analysis (FBA). The defined phases I (light pink), II (light blue), and III (light green) are indicated with background shaded colored panels. The *in silico* and *in vivo* data are shown with dotted lines and cycles, respectively. Residual biomass data (blue), PHA data (red), (*R*)-HAs (green), and residual octanoate consumption (mM, black) are shown.

To further understand the effect that increasing the flux through the PHA cycle might have on the physiology of P. putida, the experimental data collected under PHA accumulation conditions (Table 1 and Table S3) were contextualized using the metabolic model *i*JN1411. Condition-specific models were set up using a well-known step-by-step procedure (33). Interestingly, we noticed three distinct growth phases, possibly corresponding to three different steady states under PHA accumulation conditions (Fig. 4A). In phase I (between 0 and 5 h of growth; early exponential phase), the bacteria largely grew, and there was a slight PHA accumulation and/or (*R*)-HA production. In phase II (between 5 and 10 h of growth; late exponential phase), bacteria were mainly producing PHA or (*R*)-HAs, and low levels of growth were recorded. Finally, in phase III (between 10 and 24 h of growth; stationary phase), growth was either small or nonexistent and/or production of PHA/(*R*)-HA free monomers was registered. We set up three independent growth phase condition models for each strain (early exponential, late exponential, and stationary phases) to match the existence of these three consecutive steady states. Overlapping all three steady states, we were able to model the entire growth curve and define the metabolic processes taking place in each phase (e.g., PHA and free monomer production, cell growth, and octanoate consumption).

10.1128/mBio.01794-21.3 TABLE S3 Physiological data at different time points during the growth curve. They were used in the *in silico* contextualization process under PHA accumulation conditions. ND, not detected. Download Table S3, DOCX file, 0.04 MB. Copyright © 2022 Manoli et al. 2022 Manoli et al. https://creativecommons.org/licenses/by/4.0/ This content is distributed under the terms of the Creative Commons Attribution 4.0 International license.

Regarding model construction, we transformed experimental data into flux rates (mmol·g CDW^−1^·h^−1^) for compatibility and used octanoate uptake rate, growth rate, initial residual biomass, and PHA and (*R*)-HA production rates for the three different phases as model constraints for *i*JN1411. Flux balance analysis (FBA) at optimum growth levels was used to generate condition-specific model predictions. Results showed a high level of agreement with our experimental data irrespective of the strain (Fig. 4). In fact, the models accurately predicted what happens *in vivo* in terms of octanoate consumption, growth rates, and PHA and/or (*R*)-HA production. Therefore, our models are powerful computational tools to study the impact of PhaZ doses on P. putida’s metabolism under the given experimental conditions.

### Model-based phenotyping data contextualization highlights large metabolic changes on central metabolism in response to increasing flux through PHA cycle.

**FIG 5:**
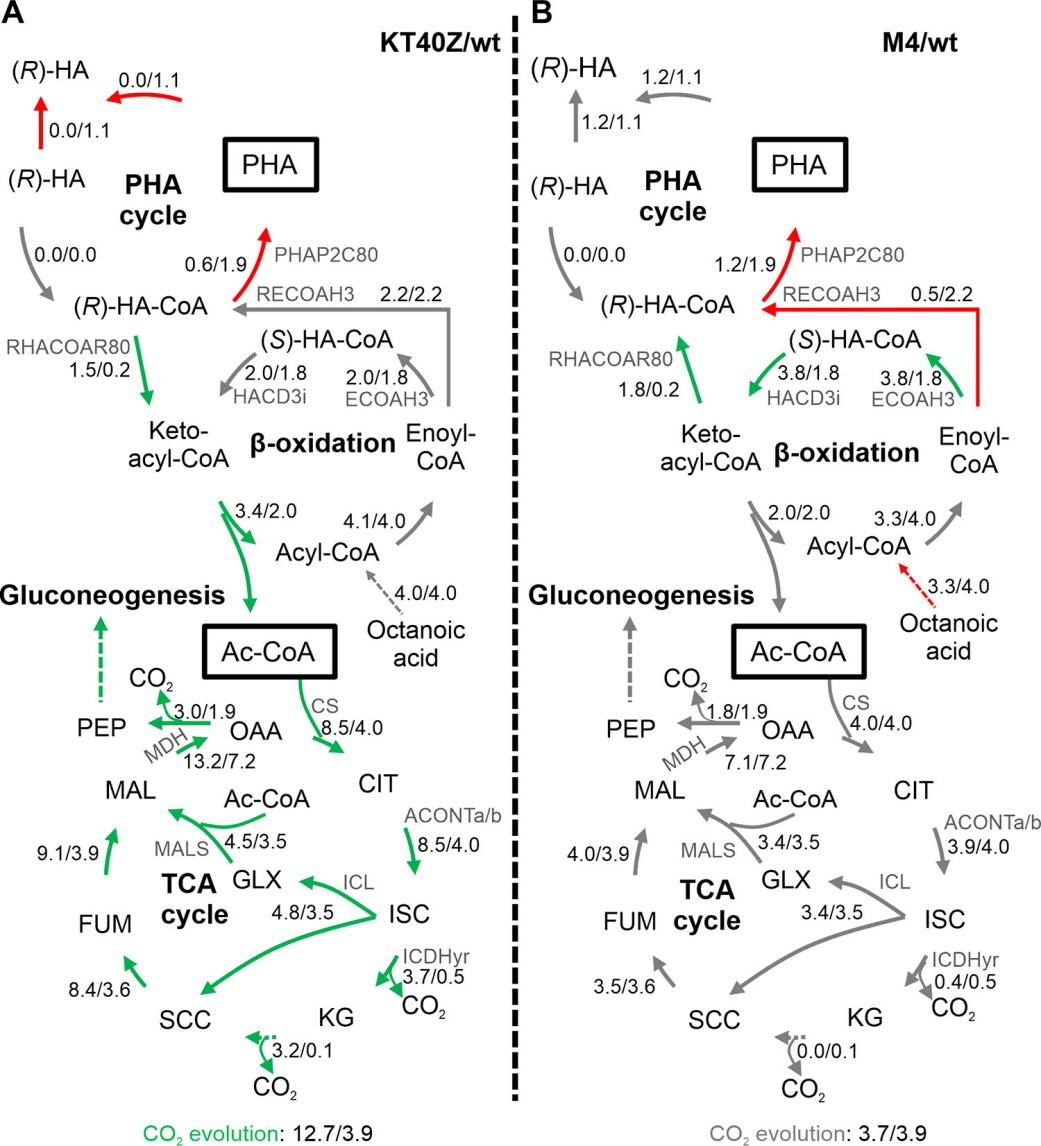
Impact of PhaZ depolymerase dosage on the distribution of central metabolite fluxes, as determined by Monte Carlo random sampling in phase I (0 to 5 h). The diagrams summarize the reaction networks in cells growing under PHA accumulation conditions. Modifications in the carbon flux resulting from the deletion (A) or the overexpression (B) of the *phaZ* gene are indicated with red arrows (reduced flux) and green arrows (increased flux), and gray arrows point to unaffected fluxes. The numbers next to the arrows indicate the net flux (mmol·gCDW^−1^·h^−1^) in the mutant and wild-type strain (mutant/wild type). The two (*R*)-HAs correspond to the intracellular and extracellular compounds. Abbreviations: Ac-CoA, acetyl-CoA; PEP, phosphoenolpyruvate; OAA, oxaloacetate; CIT, citrate; ISC, isocitrate; KG, a-ketoglutarate; SCC, succinate; FUM, fumarate; MAL; malate; GLX, glyoxylate.

To further analyze the impact of increasing levels of PhaZ on P. putida’s metabolism at the system level, the solution space in each growth phase model was randomly sampled using the Markov chain Monte Carlo approach (34). Thus, the probabilistic flux value for each reaction in the network was computed using a random set of points from the solution space as a proxy of the entire space. Results obtained for strains KT40Z and M4 and their comparison with the wild-type strain are summarized in Fig. 5 (see also Fig. 7). The carbon flux distribution predictions for the rest of the strains are listed in Data Set S1.

10.1128/mBio.01794-21.4 DATA SET S1 Predicted carbon flux distribution during phases I, II, and III. Median values for reaction fluxes (mmol·gCDW^−1^·h^−1^) were obtained using Monte Carlo random sampling for condition-specific models of phases I, II, and III. The reactions affecting key metabolic pathways, such as EMP, pay-off, ED, gluconeogenesis, PPP, TCA, key exchange reactions, PHA cycle, and fatty acid metabolism reactions are shown. Download Data Set S1, XLSX file, 0.3 MB. Copyright © 2022 Manoli et al. 2022 Manoli et al. https://creativecommons.org/licenses/by/4.0/ This content is distributed under the terms of the Creative Commons Attribution 4.0 International license.

According to model predictions, deleting the depolymerase reaction led to significant carbon flux distribution changes compared to the wild-type strain in phase I (early exponential) (Fig. 5A). As might be expected, strain KT40Z displayed a complete PHA cycle blockage due to the absence of flux through the PHA polymerase reaction (PHAP2C80). Interestingly, the PHA polymerase substrate, (*R*)-HA-CoA, was not completely incorporated into nascent PHA but instead was significantly funneled to the β-oxidation pathway through the reaction catalyzed by 3-oxoacyl-ACP reductase, FabG (RHACOAR80), and subsequently transformed into acetyl-CoA, feeding the tricarboxylic acid (TCA) cycle and oxidative metabolism.

Key reactions of the TCA cycle, including citrate synthase (CS), aconitate dehydratase (ACONTa/b), and malate dehydrogenase (MDH), resulted in 1.8 to 2.2 times higher flux than the wild-type model. Additionally, during this phase KT40Z showed a 1.3-fold higher flux through the glyoxylate shunt reactions, e.g., isocitrate lyase (ICL) and malate synthase (MALS), thus providing higher levels of C_4_ metabolites from acetyl-CoA. According to model predictions, this excess of C_4_ metabolites was rerouted to biomass building blocks and sugars via gluconeogenesis. In fact, 1.5 to 1.8 increased flux was predicted in this pathway compared to the wild-type strain (glyceraldehyde-3-P-dehydrogenase, GAPD; phosphoglycerate kinase, PGK; phosphoglycerate mutase, PGM; phosphopyruvate hydratase, ENO reactions).

Accordingly, the KT40Z model predicted 1.7-fold ATP production compared to the wild type under this high level of activity of the TCA cycle (Data Set S1). Finally, because of this high oxidative metabolism, KT40Z produced 3.2 times more CO_2_ and registered a 2.2-fold higher respiration rate than the wild-type model (Data Set S1).

On the other hand, increasing the PhaZ concentration in phase I had no major effects on either the predicted carbon flux distribution around the TCA cycle or gluconeogenesis compared to the wild-type strain (Fig. 5B). Interestingly, model-based predictions suggested different pathways providing (*R*)-HA-CoA. Hence, while 3-oxoacyl-ACP reductase (RHACOAR80) was predicted to provide (*R*)-HA-CoA in strain M4, enoyl-CoA hydratase, PhaJ (RECOAH3), acted as a major source of (*R*)-HA-CoA in the wild-type strain. Since the 3-oxoacyl-ACP reductase (RHACOAR80)-based pathway is closely assisted by enoyl-CoA dehydratase, FadB (ECOAH3), and 3-hydroxyacyl-CoA dehydrogenase and FadB (HACD3i) exchanges a mole of NADH-NADPH per mole of (*R*)-HA-CoA produced, it is tempting to speculate that this alternative pathway in strain M4 is a consequence of a putative balancing of the reducing equivalent (described below).

**FIG 6:**
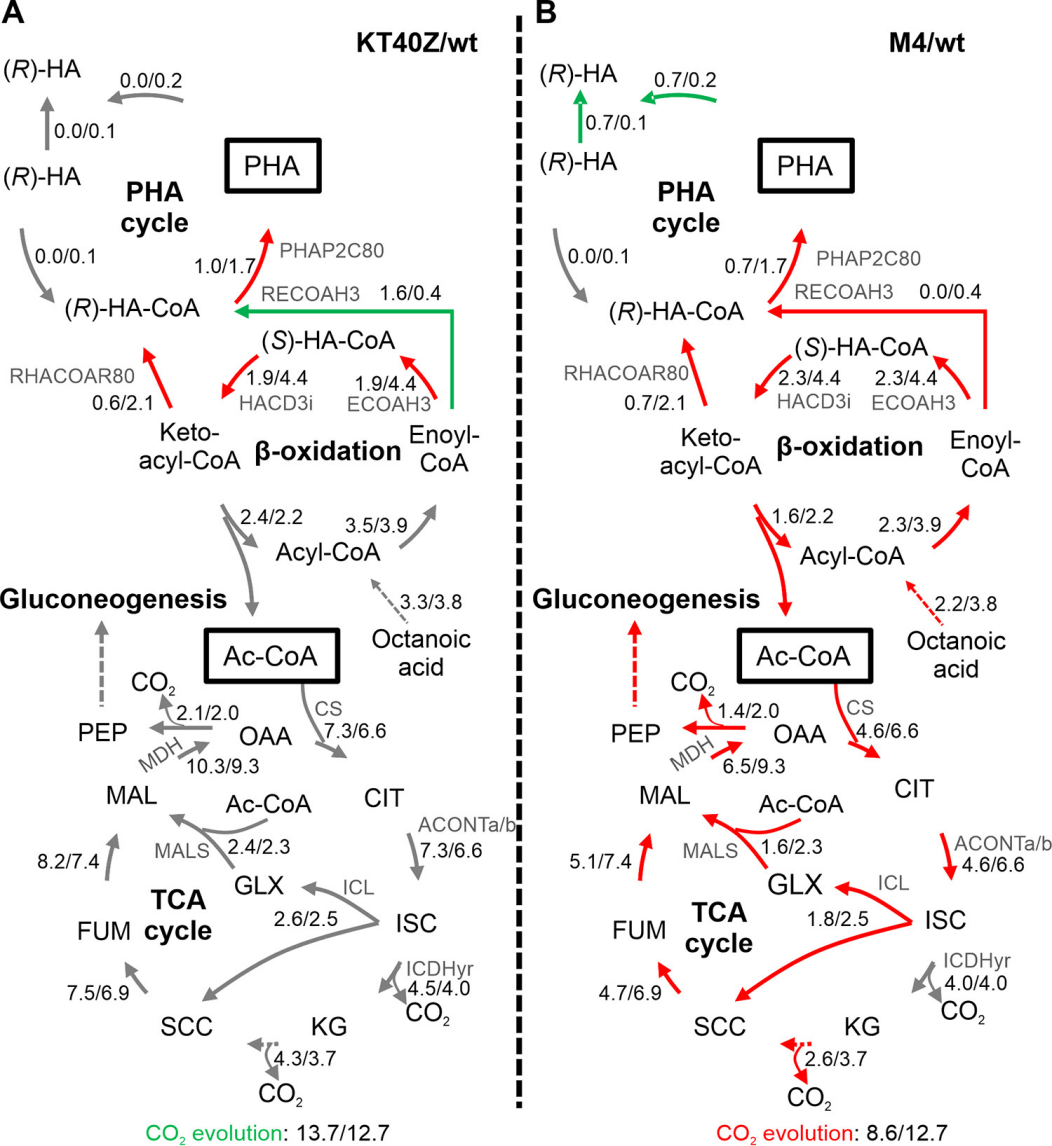
Impact of PhaZ depolymerase dosage on the distribution of central metabolite fluxes, as determined by Monte Carlo random sampling in phase II (5 to 10 h). The diagrams summarize the reaction networks in cells growing under PHA accumulation conditions. Modifications in the carbon flux resulting from the deletion (A) or the overexpression (B) of the *phaZ* gene are indicated by red arrows (reduced flux) and green arrows (increased flux), and gray arrows point to unaffected fluxes. The numbers next to the arrows indicate the net flux (mmol·g CDW^−1^·h^−1^) in the mutant and wild-type strains (mutant/wild type). The two (*R*)-HAs correspond to the intracellular and extracellular compound. Abbreviations: Ac-CoA, acetyl-CoA; PEP, phosphoenolpyruvate; OAA, oxaloacetate; CIT, citrate; ISC, isocitrate; KG, a-ketoglutarate; SCC, succinate; FUM, fumarate; MAL; malate; GLX, glyoxylate.

Concerning the predicted carbon flux distribution during phase II (late exponential), no significant differences were observed between KT40Z and the wild-type strain. However, we did register differential production of (*R*)-HA-CoA when strain KT40Z used mainly the enoyl-CoA hydratase (RECOAH3) reaction, while the wild-type strain synthesized (*R*)-HA-CoA through the 3-oxoacyl-ACP reductase (RHACOAR80) reaction (Fig. 6A and Data Set S1).

In contrast, the M4 model shows significant differences in terms of flux distribution compared to the wild-type model (Fig. 6B and Data Set S1). Overall, the model predicts an important metabolism deceleration in M4 that is likely driven by a lower uptake of octanoate (2.2 versus 3.8). Significant decreases in the flux through the β-oxidation pathway, PHA synthesis, TCA cycle, and gluconeogenesis were predicted (Fig. 6B). As expected by the high levels of depolymerase present in strain M4, all the PHA synthesized was further hydrolyzed and subsequently secreted to the medium. Interestingly, this observed metabolism deceleration had no influence on growth rate, and similar values were found in both strains. These results strongly suggest that, during this phase, the greater the PhaZ activity, the lower the carbon spilling in the form of CO_2_.

**FIG 7:**
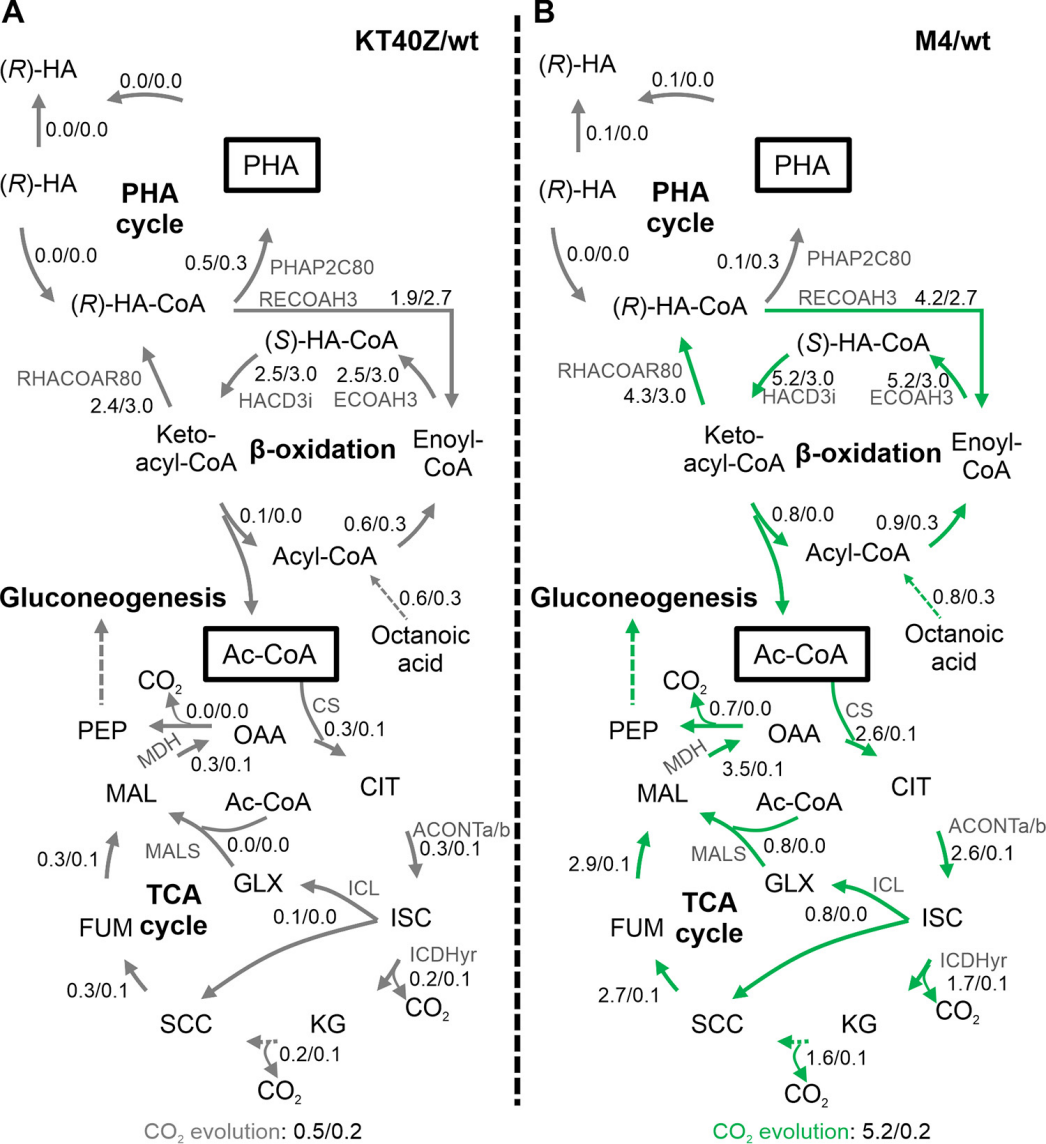
Impact of PhaZ depolymerase dosage on the distribution of central metabolite fluxes, as determined by Monte Carlo random sampling in phase III (10 to 24 h). The diagrams summarize the reaction networks in cells growing under PHA accumulation conditions. Carbon flux alterations resulting from the deletion (A) or the overexpression (B) of the *phaZ* gene are indicated by red arrows (reduced flux) and green arrows (increased flux), and gray arrows point to unaffected fluxes. The numbers next to the arrows indicate the net flux (mmol·g CDW^−1^·h^−1^) in the mutant and wild-type strain (mutant/wild type). The two (*R*)-HAs correspond to the intracellular and extracellular compound. Abbreviations: Ac-CoA, acetyl-CoA; PEP, phosphoenolpyruvate; OAA, oxaloacetate; CIT, citrate; ISC, isocitrate; KG, a-ketoglutarate; SCC, succinate; FUM, fumarate; MAL; malate; GLX, glyoxylate.

**FIG 8:**
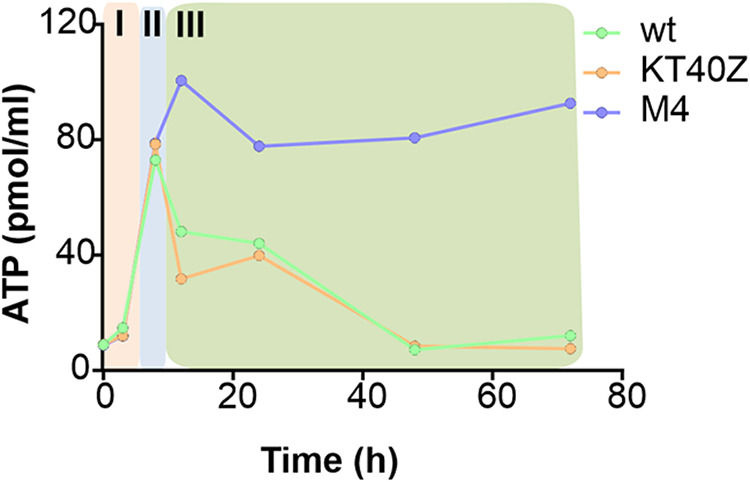
ATP quantification during the different phases. The defined phases I (light pink), II (light blue), and III (light green) are indicated with background-shaded color panels. These data relate to a single biological replicate.

Similar to what has been described for phase II, in phase III (stationary phase) carbon flux distribution predicted for the wild-type and KT40Z strains indicated that the deletion of *phaZ* had little effect on P. putida’s physiology when growing under PHA accumulation conditions (Fig. 7A and Data Set S1). In both strains, phase III was dominated by a basal metabolism driven by reduced octanoate uptake and modest activity of β-oxidation and the TCA cycle. Interestingly, for phase III, strain M4 displayed a very active metabolism that was characterized by an increased flux through β-oxidation, which in turn drove significant oxidation of acetyl-CoA in the TCA cycle. Consequently, strain M4 maintained significant levels of activity through oxidative phosphorylation that resulted in high production of ATP despite having reached the stationary phase (Fig. 7B and Data Set S1). In fact, the ATP production rate was 9.8 times higher in the M4 background strain than in the wild type. To validate this model-driven hypothesis, we monitored levels of ATP in these strains *in vivo*. Indeed, the results of our *in vivo* ATP quantification experiments are in agreement with the computational data, and we found that strain M4 contains 10 times higher ATP levels than the wild type and KT40Z strains (Fig. 8). These experiments confirmed that such high levels were maintained 72 h into the stationary phase. Similarly, CO_2_ production in the mutant strain was higher than that in the wild type, which is in accordance with *in vivo* CO_2_ production quantification (as described above) (Fig. 3E).

Based on carbon flux distribution predictions (Fig. 5 and 7 and Data Set S1), it could be concluded that the level of PhaZ depolymerase has a significant impact on the overall metabolism of P. putida. In fact, significant carbon flux alterations were observed during phase I in the absence of *phaZ*. On the contrary, increased levels of PhaZ led to minor changes during the first two phases, which indicated a metabolic deceleration. However, a significantly higher oxidative metabolism was displayed during phase III (stationary) compared to the wild-type and KT40Z strains. These results demonstrate that the carbon metabolism of P. putida could be controlled by modulating the activity of the PHA cycle, potentially supporting tailored biotechnological applications.

### Metabolic robustness and stress endurance are largely powered by the PHA cycle in Pseudomonas.

**FIG 9:**
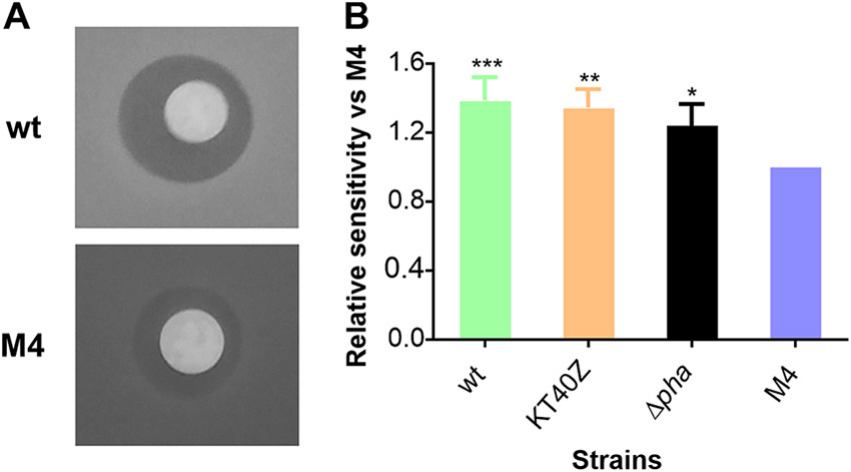
Oxidative stress assays in plate. (A) Inhibition halos for strains KT2440 and M4 after 24 h of growth under PHA accumulation conditions plus 30% H_2_O_2_ stress. (B) Relative sensitivity versus strain M4 for KT2440, KT40Z, and KT2440 Δ*pha* strains after 24 h of growth under PHA accumulation conditions. The inhibition halo area was quantified using Image J software. One-way ANOVA was performed, and significant differences compared to strain M4 are shown. Dunnett’s multiple-comparison test was applied. *, *P* ≤ 0.05; **, *P* ≤ 0.01; ***, *P* ≤ 0.001.

Aerobic microorganisms such as Pseudomonas use O_2_ for respiration or oxidation of nutrients to obtain energy. Reactive by-products of oxygen (e.g., hydrogen peroxide and superoxide anion) are generated continuously in cells growing under aerobic conditions, triggering oxidative stress. The importance of the PHA cycle in providing resistance to different environmental perturbations was previously described for several microorganisms (35, 36). With this work we specifically addressed the role of the PHA cycle as a major player in oxidative stress resistance, a well-known metabolic feature of P. putida (37). Therefore, we checked whether higher levels of PHA hydrolysis might lead to improved tolerance to oxidative stress by comparing the phenotype of M4, KT40Z, and the wild-type strains. This was done using standard oxidative stress assays in plates under PHA accumulation conditions. Detailed analysis of the relative sensitivity of strain M4 versus the control strains revealed that there is a correlation between higher resistance to oxidative stress and a higher PHA hydrolysis rate. Indeed, strain M4 exhibited 1.3 to 1.5 times more tolerance to oxidative stress than the other strains tested (Fig. 9). Since high levels of NADPH are associated with higher tolerance to oxidative stress (38), these results are consistent with the model prediction, suggesting higher levels of NADPH associated with higher PhaZ activity (Fig. 7B). Furthermore, the level of active metabolism predicted for the stationary phase is also compatible with the higher stress endurance observed in the oxidative stress assays.

## DISCUSSION

Apart from its primary carbon storage function, PHA metabolism has been defined as a buffering cycle regulating global biomass, cell division, and energy spillage (19, 22, 23). Thus, to contribute to our holistic understanding of the PHA cycle’s impact on Pseudomonas physiology, we have followed a multidisciplinary approach combining advances in synthetic and systems biology. We first inhibited PHA cycle turnover in P. putida cells by deleting the *phaZ* gene while maintaining the functionality of the native PHA production machinery (polymerases and phasins). We then generated and validated a battery of strains displaying differential *phaZ* expression to help fine-tune PHA hydrolysis. Finally, we generated a large set of phenotyping data from these strains and framed them in the context of condition-specific metabolic models. This allowed us to decipher the metabolic features driven by PHA cycle activity in Pseudomonas.

### Tuning PHA hydrolysis strongly powers metabolic shifts and robustness in bacteria.

Bacterial response to sudden environmental changes has been thoroughly studied at the regulatory level (39). However, the metabolic process powering such a highly demanding shift in terms of ATP and reducing equivalent requirements is still poorly understood. Here, we show that by increasing the flux through PhaZ, the PHA hydrolysis process is promoted, leading to impaired PHA accumulation and an increase in secretion of free monomers to the supernatant. This prevents fatty acids from being fully catabolized; instead, cells release metabolic intermediates [(*R*)-HAs] as a preprocessed and readily mobilized carbon store. Meanwhile, P. putida reorients its metabolism toward the generation of surplus energy that remains available for transfer to alternative processes (described below). The retained free (*R*)-HAs can be redirected to the β-oxidation pathway if nutrient limitation is overcome (e.g., nitrogen limitation in this work). Higher fluxes through PhaZ also resulted in a higher cell division rate, as was concluded from observations of larger cell numbers compared with the wild-type and KT40Z strains. The smaller size of viable cells can be explained by a combination of high division rate and the limitation of growth conditions by nitrogen availability, which drives and limits the formation of residual biomass. Overall, we provide here solid evidence that by increasing the rate of PHA hydrolysis along an engineered PHA cycle, cells can control levels of carbon and energy in order to face environmental disturbances, such as oxidative stress.

Extracellular release of (*R*)-HAs in response to higher PHA turnover confers additional benefits to bacteria that go beyond providing an extra carbon source. For instance, they may also act as antimicrobial, insecticidal, and antiviral agents (40–42). In other species, like *Methylobacterium*, Koskimäki and collaborators demonstrated that methyl-esterified 3HB oligomers could protect bacteria from hydroxyl radicals (42). Recently, it has also been pointed out that 3-HA reduction is an important adaptation mechanism during sustained, industrial-scale starvation conditions (43). Besides the role of (*R*)-HAs, several studies have also reported the relationship between PHA and bacterial stress resistance in harsh environments such as plant colonization (44), and PHA hydrolysis can also benefit non-PHA producers (45).

Altogether, it is reasonable to assume that the metabolic robustness of pseudomonads and other bacterial groups can be controlled to a great extent by tuning PHA hydrolysis, which in turn fuels multiple metabolic processes such as response to environmental disturbances and bacterial persistence during infection in the case of pathogenic strains.

### Cell physiological changes correlate with different metabolic states dependent on PhaZ levels.

Pseudomonads are well-known for their metabolic versatility (46). They can use different carbon sources and rewire their central metabolism to deal with cell requirements such as energy conservation and proliferation under harsh environmental conditions (3, 47). To test whether the flux through the PHA cycle could be responsible, at least in part, for these metabolic shifts, we used the genome-scale *i*JN1411 metabolic model as a computational scaffold for data analysis at the system level. We identified and analyzed metabolic fluxes under three different metabolic states: growth (I), PHA or (*R*)-HA accumulation (II), and stationary phase (III). Despite metabolic flux analysis, using a stoichiometric kinetic model, having been previously performed during the pseudo-steady-state period (between 4 and 8.5 h of growth) (22), we go beyond that here by providing a reconstruction of the dynamic carbon flux readjustment along the entire growth curve. We address each of the steady phases, which is in accordance with the determination of different cell fates in the exponential, stationary, and long stationary phases (48). Stationary-phase survival is a strategy for cell adaptation to conditions of stress or starvation. During this phase, the bacterium exhibits several changes concerning its morphology, metabolism, and transcriptional and translational levels. This is the case in several bacterial groups, including pseudomonads. For instance, metabolic changes have been observed during P. aeruginosa infection, mostly affecting the flux through the glyoxylate shunt and TCA cycle (49). Here, we show that endurance over the stationary phase is likely fueled, at least in part, by higher PHA hydrolysis.

### PHA hydrolysis beyond metabolic shift facilitator.

Beyond the PHA-driven control over metabolism in Pseudomonas, the availability of P. putida strains displaying high flux through PhaZ provides great potential for biotechnological applications. The (*R*)-HA monomers are valuable substrates because they can be used as building blocks for a variety of biotechnological applications, such as synthesis of antibiotics, polyesters, and vitamins (40, 50, 51). Key interrelated aspects to consider for cost-effective, large-scale (*R*)-HA production are substrate cost, product purification, and strain fitness (50–52). In this work, we provide a set of engineered P. putida strains producing differential amounts of (*R*)-HAs that are also higher than (*R*)-HAs produced by the wild type. This is achieved under the control of a constitutive promoter and without the need for an external inductor, which is an important aspect to consider for subsequent upscaling. So far, it can be inferred that M2 to M4 strains are promising candidates for large-scale production of enantiopure HAs and other biotechnological applications (48, 53).

Overall, our study suggests that different metabolic states in Pseudomonas can be engineered by tailoring the flux through the PHA cycle, providing promising features concerning environmental stress resistance and subsequent biotechnological applications.

## MATERIALS AND METHODS

### Bacterial strains, media, and growth conditions.

Bacterial strains and plasmids used in this work are listed in Table S1 in the supplemental material. E. coli and P. putida strains were grown routinely for DNA manipulations and for precultures in lysogeny broth (LB) medium at 37°C and 30°C, respectively (54). Suitable antibiotics, i.e., gentamicin (10 μg/ml), chloramphenicol (34 μg/ml), and kanamycin (50 μg/ml), were added when needed.

For PHA accumulation experiments, P. putida strains were grown in 0.1N M63 nitrogen-limited minimal medium [13.6 g of KH_2_PO_4_/L, 0.2 g (NH_4_)_2_SO_4_/L, 0.5 mg FeSO_4_×7 H_2_O/L, adjusted to pH 7.0 with KOH], supplemented with 15 mM sodium octanoate (C/N ratio, 40 mol/mol) for 24 h at 30°C under vigorous shaking at 200 rpm (19). The medium was supplemented with 1 mM MgSO_4_ and a 1× solution of trace elements (Goodies) (composition, 1,000× dissolved in 1N HCl: 2.78 g/L FeSO_4_×7H_2_O, 1.98 g/L MnCl_2_×4H_2_O, 2.81 g/L CoSO_4_×7H_2_O, 1.47 g/L CaCl_2_×2H_2_O, 0.17 g/L CuCl_2_×2H_2_O, 0.29 g/L ZnSO_4_×7H_2_O).

### Construction of P. putida KT40Z deletion mutant.

Standard molecular biology techniques were used as previously described (54). The *phaZ* gene (PP_5004) was inactivated by allelic exchange homologous recombination using the mobilizable plasmid pK18*mob*sacB (55). PCR primer pairs (Table S2) were designed to amplify approximately 800-bp regions upstream (Z1) and downstream (Z3) of the *phaZ* gene to serve as recombination arms of homology. Restriction enzyme sites were added upstream of Z1 (XbaI) and downstream of Z3 (HindIII-HF) to clone the fragment Z1Z3 into pK18*mobsacB*. The resulting pK18*mobsacB*-Z1Z3 construction was inserted in P. putida KT2440’s genome using the triparental filter-mating technique (56). For the first recombination selection process, cetrimide agar plates supplemented with 50 μg/ml kanamycin were used, allowing only the selection of Pseudomonas strains (57). The resulting recombinant strains were confirmed by PCR, and selected colonies were grown in LB for 6 h and then transferred to M63 minimal medium plus 10 mM citrate selective plates supplemented with 5% sucrose. Transconjugants, sucrose resistant and kanamycin sensitive, were isolated, and the second recombination event was confirmed by PCR using external primers of the arms of the homology region and DNA sequencing (Table S2).

### Construction of a library of strains harboring differential *phaZ* levels.

The synthetic promoter library previously developed in P. putida KT2440 (57) was used to obtain E. coli constructs driving differential *phaZ* expression levels. The pBG-derived version plasmids contained three modules: the synthetic promoter (only variable), the translational coupler or bicistronic element BCD2, and the reporting gene *msf-gfp* (57). Based on pBG’s derived structure, BCD2-*msf*-*gfp* was replaced with BCD2-*phaZ*. The latter construct was obtained using PCR amplification of the P. putida genome with AvrII-BCD2-*phaZ* and *phaZ-*BamHI-Rev primers using Phusion polymerase (Table S2). The BCD2-*phaZ* construct was subcloned into the pBG-derived vectors using AvrII and BamHI-HF restriction enzymes, resulting in a panel of pBG-derived vectors expressing the *phaZ* gene at different doses (Table S1). Genome integration of these constructs into P. putida KT40Z was performed using the mini-Tn*7* transposon. The resulting strains that should theoretically account for increasing levels of *phaZ* expression were named M0 to M4, where M0 had the lowest and M4 the highest promoter strength (Table S1).

### RNA extraction and qRT-PCR experiments.

Standard molecular biology techniques were followed to extract RNA (22). The RNA samples were obtained from at least three independent cultures grown under PHA accumulation conditions. After 6 h of growth (at mid-exponential phase), 7 ml was harvested by centrifugation at 3,000 × *g* for 10 min at 4°C. Cell pellets were rapidly frozen in dry ice and stored at −80°C until further use. Pellets were resuspended in TE buffer (10 mM Tris-HCl, pH 7.5, 1 mM EDTA) containing 5 mg/ml lysozyme. RNA was extracted using the High Pure RNA isolation kit (Roche) by following the manufacturer’s instructions. Extracted RNA was additionally treated with DNase (Ambion) by following the manufacturer’s instructions. RNA integrity was checked by agarose gel electrophoresis and quantified with a NanoDrop 2000 spectrophotometer (Thermo Scientific, MA, USA).cDNA synthesis was performed using the Transcriptor first-strand cDNA synthesis kit (Roche) by following the manufacturer’s recommendations. cDNA was synthesized from 1 μg of purified RNA using random hexamer-primed reactions. For RT-PCR, 1 μg of transcribed cDNA was used and a standard curve of differential dilutions (from 10^−1^ to 10^−5^) of P. putida genomic DNA was plotted. Primer sequences used in this study are listed in Table S2. Data were analyzed using absolute quantification to show expression levels as a concentration of cDNA (nanomolars). This analysis was performed in three technical replicates from three independent biological samples, and the size of each amplified gene was considered.

### Physiological studies and calculation of parameters.

For P. putida growth experiments, LB preculture cells were washed twice with 0.85% saline solution and adjusted to an OD_600_ of 0.3 at 600 nm in a PHA accumulation-favoring medium. Culture growth (50 ml) was monitored in shaking 250-ml Erlenmeyer flasks (at 200 rpm) using a portable spectrophotometer (Fisher Scientific, PA, USA) at 600 nm for 24 h.

For total biomass calculations, standard assays were performed as previously described (19). Briefly, 40 ml of culture medium was centrifuged in previously tared 50-ml Falcon tubes for 45 min at 3,000 × *g* at 4°C. Cell pellets were rapidly frozen at −80°C and freeze-dried for 24 h in a lyophilizer. Finally, the tubes were weighed and cell densities were expressed as grams of cell dry weight (CDW) per L. For viable cell number calculations, we followed reference 19.

Given that PHA content affects cell turbidimetry, optical density returns mixed information about cell growth and PHA production and therefore cannot be used to calculate growth rate. Differences in cell size and the number of viable cells over time cannot be used for this purpose either. Therefore, log_10_ of residual biomass (biomass free of PHA) data versus time was used to calculate growth rate. The curve’s slope was multiplied by a conversion factor (2.303) between Napierian logarithms.

### *In vivo* determination of CO_2_ production.

*In vivo* determination of CO_2_ production was carried out using MicroResp (patent GB2410797; Macaulay Scientific Consulting, Aberdeen, UK) as previously described (22). A 900-μl aliquot of P. putida batch culture grown for 24 h under PHA accumulation conditions was placed in every well of a 1.2-ml deep-well plate. A second plate was used as a detection system for evolved carbon dioxide (58). This was sealed to the culture plate using a silicone rubber gasket with perforations to allow connectivity between corresponding wells. Detection wells were filled with a solution of Cresol Red indicator dye (0.033 mM), potassium chloride (150 mM), and sodium bicarbonate (2.5 mM) mixed with 150 μl of purified agar (1%). Purified agar was used due to its lower absorbance and because it does not affect the wavelength of the indicator dye. Detection plates were stored at room temperature in the presence of soda lime to ensure desiccation and avoid contamination with atmospheric CO_2_. Plate assemblages were incubated at 30°C with shaking at 100 rpm, and evolved CO_2_ was measured by means of colorimetric analysis at 0 h and immediately after 6 h of incubation. Readings were taken from detection plates using a microtiter plate reader (Thermo Scientific Varioskan flash) at 570 nm, which is the optimum wavelength for the indicator dye.

The MicroResp system requires calibration at each laboratory to consider the unique features of the spectrophotometer being used, the different types of environmental samples, and incubation conditions. A calibration curve of dye color against CO_2_ concentration was plotted as previously described by our group (22), and a single rectangular hyperbola was obtained: *y* = (*A · x*)/(*B + x*), *R*^2^ = 0.9713, where *y* is the %CO_2_, *x* the OD_570_, *A* is 1.73, and *B* is −0.13.

### Oxidative stress assays.

Bacterial response to oxidative stress induced by hydrogen peroxide was measured as described previously by Chavarria and collaborators, with some modifications (59). Briefly, LB preculture cells were inoculated in fresh LB medium and set to an OD_600_ of 0.5 to 0.6. The cells were then washed twice with 0.85% saline solution and concentrated 10-fold. Culture plates were prepared using the top lid of dishes with 0.1N M63 medium supplemented with 15 mM octanoate (PHA accumulation conditions). A volume of 1 ml of cells was added to a less concentrated agar (0.7% soft agar) with the same medium composition and poured onto the plates. When the top agar was solidified, homogeneous filters (Whatman qualitative filter paper, grade 1) were placed in the middle of the plate, and 5 μl of 30% H_2_O_2_ was added. The plates were incubated at 30°C for 24 h and then photographed. The halo inhibition area was quantified using Image J software. At least three technical and biological replicates were carried out. The data were normalized to M4 sensitivity.

### Intracellular ATP measurements.

Intracellular ATP levels were determined using an ATP bioluminescence assay kit (ATP biomass kit HS; Biothema, Sweden) per the manufacturer’s instructions. To measure intracellular ATP, 1 ml of P. putida cells was centrifuged for 1 min at 13,000 × *g* and 4°C, and the pellet was resuspended in 1 ml of saline solution (0.85% NaCl) to remove any extracellular ATP.

### Methanolysis process and GC-MS analysis for PHA determination.

For composition and total cellular PHA content quantification, standard gas chromatography-mass spectrometry (GC-MS) approaches of the methanolyzed polyester were used (19). Briefly, 2 to 5 mg of lyophilized samples (culture pellets) was resuspended in 2 ml of methanol containing 15% sulfuric acid and 2 ml of chloroform containing 0.5 mg/ml 3-methylbenzoic acid (3MB) as an internal standard and then incubated using a screw-cap tube at 100°C for 5 h. After cooling, 1 ml of distilled water was added to the mixture to extract most cell debris and any remaining sulfuric acid. A two-phase extraction process was performed to completely remove the water phase to prevent fouling of the GC column. Finally, a small amount of Na_2_SO_4_ powder was added to dry the chloroform phase and to remove any remaining water. The organic phase containing the resulting methyl esters of monomers was analyzed by GC-MS.

An Agilent (Waldbronn, Germany) series 7890A gas chromatograph coupled with a 5975C MS detector (EI; 70 eV) and a split–splitless injector were used for the analyses. An aliquot (1 μl) of organic phase was injected into the gas chromatograph at a split ratio of 1:50. For this work, a DB-5HTDB-5HT column (400°C; 30 m by 0.25 mm by 0.1-μm film thickness) was used. Helium was used as the carrier gas at a flow rate of 0.9 ml/min. The injector and transfer line temperature were set at 275°C and 300°C, respectively. For efficient peak separation, the oven temperature program was set to start at 80°C for 2 min and then rise to 175°C at a rate of 5°C min^−1^. EI mass spectra were recorded in full scan mode (*m/z* 40 to 550). The retention time for each methyl ester monomer obtained in this work was 3.5 min (C_6_), 7.2 min (C_8_), and 6.1 min (3MB; internal standard).

### Octanoate consumption quantification using GC-MS.

To quantify extracellular octanoate using GC-MS, the lyophilized supernatants of the strains growing under PHA accumulation conditions were derivatized. Approximately 5 mg of lyophilized sample was weighed, and 100 μl of pyridine and 50 μl of *N´N*´BSTFA [bis(trimethylsilyl)trifluoroacetamide] was added. The mixture was incubated for 45 min in a sand bath at 70°C with agitation. A volume of 50 μl of 10 mM *n*-decane dissolved in pyridine was then added to the mixture as an internal standard. A standard curve of sodium octanoate was plotted using the same procedure (0 to 15 mM octanoate). An HP-5MS 5% phenylmethyl Silox (400°C; 30 m by 0.25 mm by 0.1-μm film thickness) column was used. The transfer line temperature was set at 280°C. The oven temperature program was set to a starting temperature of 80°C for 0 min and then from 20°C/min up to 200°C for 0 min. For efficient separation of peaks, the overall duration of the run was 6 min. Retention time was 2.4 min and 4 min for the internal standard (decane) and octanoate, respectively.

### Extracellular (*R*)-HA content quantification using HPLC-MS.

P. putida strains were cultivated under PHA accumulation conditions. At different time points, 40 ml of culture medium was centrifuged for 45 min at 3,000 × *g* at 4°C in previously tared 50-ml Falcon tubes. Supernatants were rapidly frozen at −80°C and freeze-dried for 72 h in a lyophilizer. The tubes were later weighed for further calculations. The lyophilized supernatant was homogenized and further resuspended in a methanol-water solution (50%, vol/vol) at 10 mg/ml; 25 μl of this mixture was injected into the chromatographic system for determination of (*R*)-HA (free monomer) content using a Finnigan Surveyor pump coupled to a Finnigan LXQ TM ion trap mass spectrometer (HPLC-MS) (Thermo Electron).

Separation was performed using a 2.1- by 150-mm (3.5-μm particle size) XTerra MS C_18_ column (Waters) at a flow rate of 100 μl/min and an injection volume of 25 μl. The mobile phase was 0.1% ammonium hydroxide in water (A), 0.1% ammonium hydroxide in methanol (B), and 0.1% ammonium hydroxide in acetonitrile (C). The elution program was set as the following: at the onset, 95% A and 5% B; after 3 min, the percentage of B was linearly increased to 95% over 20 min, kept constant for 5 min, and, after that, percentage of C was increased from 0% to 45% to clean the column. Finally, it was ramped to the original composition over 5 min and then balanced for 10 min. Samples were introduced into the electrospray ionization (ESI) source in negative mode by continuous infusion using the instrument’s syringe pump at a rate of 3 ml/min. The source was operated at 4.5 kV, and the capillary temperature was set to 200°C. All spectra were recorded in full scan mode (*m/z* 50 to 1,500).

A standard curve of commercial 3-hydroxyoctanoic acid (HO; Sigma-Aldrich, Merck, Germany) was used, and we observed a deprotonated HO monomer (*m/z* 159) at a retention time of 15.4 min, a dimer adduct of HO-HO (*m/z* 603) at a retention time of 21.5 min, a trimer adduct of HO-HO-HO (*m/z* 887) at a retention time of 29.7 min and a tetramer adduct of HO-HO-HO-HO (*m/z* 1,171) at a retention time of 35.6 min (32). Sample analysis revealed two major peaks, i.e., a deprotonated HO monomer (*m/z* 159) and a dimer adduct of HO-HO (*m/z* 603).

### Identification of extracellular metabolites using HPLC.

A standard HPLC approach was used (60) to detect extracellular metabolites. Reference compounds used in this work were fructose, acetate, citrate, succinate, pyruvate, propionate, malate, formate, fumarate, oxoalacetate, ketoglutarate, butyrate, glucose, and sucrose (Sigma-Aldrich, Merck, Germany). All compounds were quantified using an Agilent Series 1260 Infinity II (Agilent, CA, USA) HPLC on an Aminex HPX-87H column (Bio-Rad, Hercules, CA, USA) at 40°C with a 0.5-ml/min flow rate and a 25-μl injection volume. The mobile phase was 2.5 mM H_2_SO_4_ applied on an isocratic regimen, and compounds were detected by means of a refractive index detector. Retention times for the aforementioned compounds were the following: sucrose (8.4 min), ketoglutarate (8.6 min), citrate (8.9 min), malate (9.5 min), oxoalacetate (9.6 min), pyruvate (9.9 min), glucose (9.9 min), fructose (10.9 min), succinate (13.4 min), formate (15.7 min), fumarate (15.8 min), acetate (17.3 min), propionate (20.4 min), and butyrate (25.8 min).

### Analyses of PhaZ production.

LB precultures of P. putida strains driving differential PhaZ production levels were grown to an OD_600_ of 0.6 in fresh LB medium at 30°C under vigorous shaking (200 rpm). Immunological techniques were applied to whole-cell extracts to determine PhaZ production levels. Western blot analysis was performed as previously described (32). Briefly, the primary anti-PhaZ antibody (1:5,000) previously washed overnight at 30°C with shaking set at 200 rpm was used with the sonicated KT40Z strain (32). A commercial anti-rabbit (1:10,000) was used as a secondary antibody (GE Healthcare). The expected PhaZ size was 31 kDa. The Western blot signal intensities were quantified using Image J software, and the resulting intensities were normalized to the M4 strain considering the OD_600_ equivalent load of each sample.

### Microscopy assays.

Cultures were routinely visualized with a 100× phase-contrast objective (Nikon microscope) and images taken with an attached camera (Leica DFC345 FX). Forty individual cells from at least three individual experiments were size quantified at different time points along the bacterial growth curve using Image J software.

For transmission electron microscopy (TEM) assays, P. putida cells previously grown under PHA accumulation conditions for 24 h were harvested and washed twice in 1× phosphate-buffered saline (PBS). The protocol previously implemented by our laboratory (19) was subsequently applied to sample staining and further processing for TEM image acquisition.

### Constraint-based flux analysis and *in silico* data contextualization.

We based our constraint-based flux analysis on previous work (33, 61). Briefly, *i*JN1411 was analyzed using the COBRA Toolbox v2.0 within the MATLAB environment (The MathWorks Inc.). The constraint-based model consists of a 2,087 by 2,826 matrix containing all stoichiometric coefficients in a model comprising 2,087 metabolites and 2,826 reactions (S). FBA was used to predict growth and flux distributions (62).

Experimental data for *in silico* data contextualization were collected from our strain growth assays under PHA accumulation conditions. The variables used were residual biomass, growth rate, octanoate uptake rate, extracellular (*R*)-HA production, and PHA production rate. Different condition-specific models were obtained and validated using FBA and dFBA analysis. For carbon flux prediction, we used Monte Carlo random sampling for each of the condition-specific models. Mixed fractions of 0.53 to 0.58 were obtained for all condition-specific models, which suggests that the solution space for these models was uniformly sampled (34). The median value from the carbon flux distribution was used as the most probable flux value.
