# Supplementary figures and images for: Synthetic Control of Metabolic States in Pseudomonas putida by Tuning Polyhydroxyalkanoate Cycle
Source: mBio. 2022 Jan 18;13(1):e01794-21. doi: 10.1128/mbio.01794-21 (PMC8764540; doi:10.1128/mbio.01794-21)

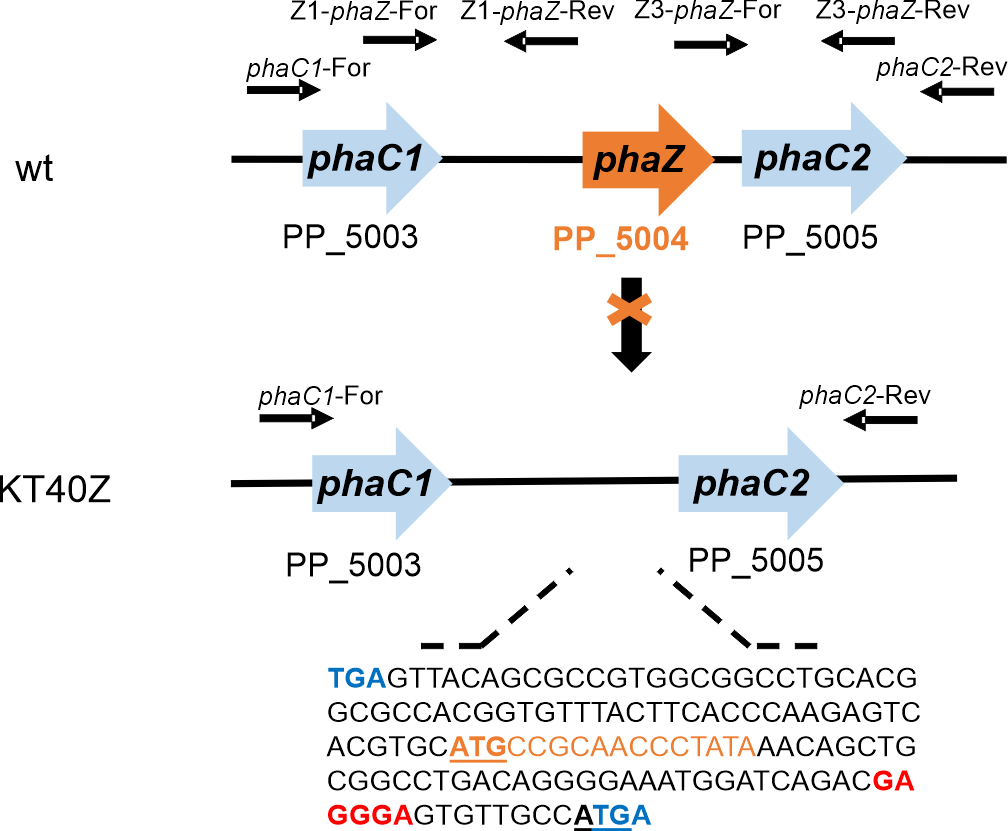

Supplement: FIG S1 [file mbio.01794-21-sf001.tif]

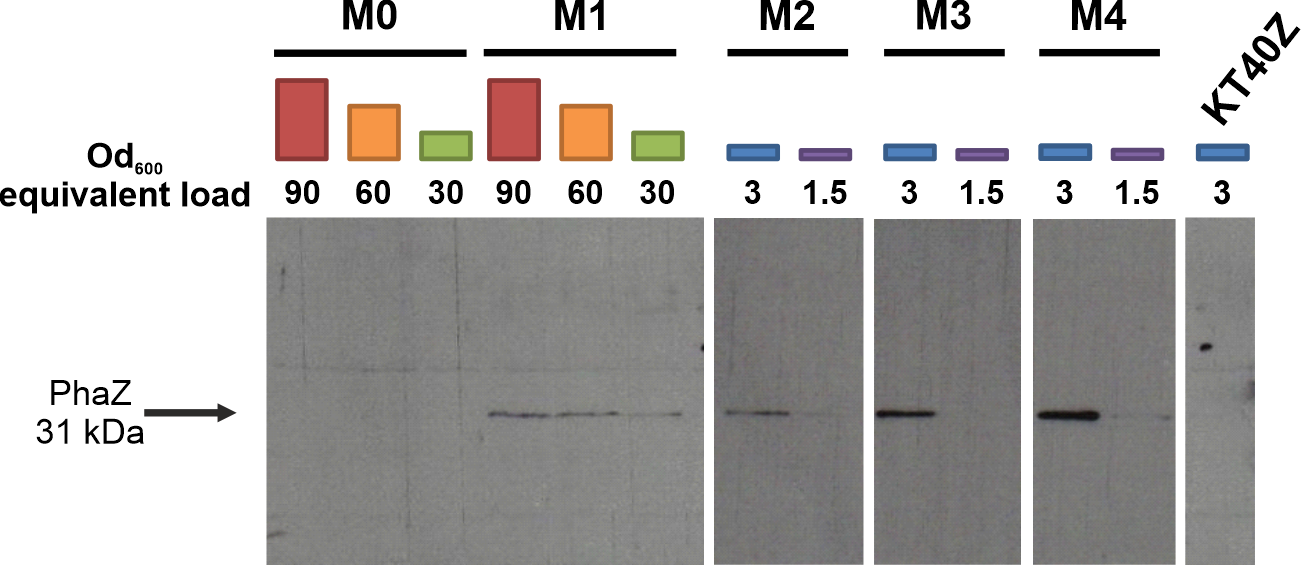

Supplement: FIG S2 [file mbio.01794-21-sf002.tif]
