# Supplementary material for: Synthetic Control of Metabolic States in Pseudomonas putida by Tuning Polyhydroxyalkanoate Cycle
Source: mBio. 2022 Jan 18;13(1):e01794-21. doi: 10.1128/mbio.01794-21 (PMC8764540; doi:10.1128/mbio.01794-21)
Supplement: TABLE S2 [file mbio.01794-21-st002.docx]

| Oligonucleotide | Sequence (5´-3´) | Utility | Genomic locus |
| --- | --- | --- | --- |
| AvrII-BCD2-*phaZ* | CCTAGGGCCCAAGTTCACTTAAAAAGGAGATCAACAATGAAAGCAATTTTCGTACTGAAACATCTTAATCATGCTAAGGAGGTTTTCTAATGCCGCAACCCTATATTTTC | To obtain BCD2-*phaZ* fusion | PP_5004 |
| *phaZ*-BamHI-Rev | GCTGTTGGATCCTCACCCCCCCGAGGCCG | To obtain BCD2-*phaZ* fusion |  |
| pBG-For | CAAGGTTCTGGACCAGTTGCG | Sequencing pBG plasmids | - |
| pBG-Rev | GGTTTTCCCAGTCACGACGC | Sequencing pBG plasmids | - |
| pBGXX-Sec | CCCGAGGCATAGGCTGTAC | Sequencing pBG plasmids | - |
| pBG-Sec-Rev | GGCAACCGAGCGTTCTGAAC | Sequencing pBG plasmids | - |
| 5-Pput-glmSUP | AGTCAGAGTTACGGAATTGTAGG | Verification of Tn*7* insertion | - |
| 3-Tn7L | ATTAGCTTACGACGCTACACCC | Verification of Tn*7* insertion | - |
| Z1-*phaZ*-For | CCTCTAGAACCCTGCTGGTCAGCGTGCTGGAC | Cloning PCR | PP_5004  Deletion |
| Z1-*phaZ*-Rev | GATCCATTTCCCCTGTCAGGCCGCAGCTGTTTATAGGGTTGCGGCATGC | Cloning PCR |  |
| Z3-*phaZ*-For | AACAGCTGCGGCCTGACAGGGGAAATGGATC | Cloning PCR |  |
| Z3-*phaZ*-Rev | CCAAGCTTAGCCGCTTTTGAGCATGTACTGGACGAAGC | Cloning PCR |  |
| *phaC1*-For | GCATCACTTGTACCGCACTGG | Sequencing primer, Genome PCR verification |  |
| *phaC2*-Rev | ATTCACGGTGGCGTGGGT | Sequencing primer, Genome PCR verification |  |
| MM170 | GCTTCTCGATCTGCTTGGTCA | qRT-PCR_*phaF* gene | PP_5007 |
| MM171 | AAGATGTTGCCGAGACTGCC | qRT-PCR_*phaF* gene |  |
| MM168 | TTGTCGAGTTGAATTTCTACCTTGC | qRT-PCR_*phaI* gene | PP_5008 |
| MM169 | CAGGAAGGCGCTGACTACTT | qRT-PCR_*phaI* gene |  |

**Table S 2.** **Oligonucleotides used in this study.** The site for restriction enzymes is underlined. The following restriction enzymes were used: AvrII (CCTAGG), BamHI-HF (GGATCC), HindIII-HF (AAGCTT) and XbaI (TCTAGA).
