# Supplementary material for: Synthetic Control of Metabolic States in Pseudomonas putida by Tuning Polyhydroxyalkanoate Cycle
Source: mBio. 2022 Jan 18;13(1):e01794-21. doi: 10.1128/mbio.01794-21 (PMC8764540; doi:10.1128/mbio.01794-21)
Supplement: TABLE S1 [file mbio.01794-21-st001.docx]

| Strains/Plasmids | Genotype/ Relevant characteristics | Reference |
| --- | --- | --- |
| ***P. putida*** | | |
| KT2440 | TOL plasmid-cured, spontaneous restriction deficient derivative of *P. putida* mt-2 | (1) |
| KT40Z | KT2440 derivative strain, Δ*phaZ* deletion mutant | This work |
| KT2440 Δ*pha* | KT2440 derivative strain, Δ*pha* cluster deletion mutant | CECT 30020 |
| M0 | Gm^r^, *P. putida* KT40Z, with genomic insertion via Tn*7* using pMM5 | This work |
| M1 | Gm^r^, *P. putida* KT40Z, with genomic insertion via Tn*7* using pMM8 | This work |
| M2 | Gm^r^, *P. putida* KT40Z, with genomic insertion via Tn*7* using pMM14 | This work |
| M3 | Gm^r^, *P. putida* KT40Z, with genomic insertion via Tn*7* using pMM11 | This work |
| M4 | Gm^r^, *P. putida* KT40Z, with genomic insertion via Tn*7* using pMM12 | This work |
|  | | |
| ***E. coli*** | | |
| DH10B | Cloning host; F-, *mcrA* Δ(*mrr hsdRMS-mcrBC*) Φ80d*lac*ΔM15 Δ*lacX74 deoR recA1 araD139* Δ(*ara-leu*)7697 | Invitrogen, Thermo Fisher Scientific, USA |
| CC118λ*pir* | Rf^r^, Sp^r^ Cloning host; Δ*(ara-leu), araD,* Δ*lacX74, galE, galK, phoA20, thi-1, rpsE, rpoB, argE* (*Am*), *recA1,* lysogenized with λ*pir* phage | (2) |
| HB101 | Helper strain; F^-^ λ^-^ *hsdS20(r_B_^-^ m_B_^-^) recA13* *leuB6*(Am) *araC14* Δ(*gpt-proA*)62 *lacY1 galK2*(Oc) *xyl-5 mtl-1 rpsL20*(Sm^R^) *glnX44*(AS) | (3) |
| DH5αλ*pir* | Cloning host; DH5α lysogenized with λ*pir* phage | (4) |
|  |  |  |
| **Plasmids** |  |  |
| pRK600 | Cm^r^, ColE1 *oriV* RK2 Mob^+^ Tra^+^ donor of transfer functions | (5) |
| pK18*mobsacB* | Km^r^, pMB1, *oriV*, Mob^+^, *lacZa*, *sacB*; vector for allelic exchange homologous recombination mutagenesis | (6) |
| pMM4 | Km^r^, pK18*mobsacB* derivative vector used for *phaZ* deletion | This work |
| pTnS-1 | Amp^r^, ori R6K, TnSABC+D operon | (7) |
| pBG | Km^r^ Gm^r^, ori R6K, Tn7L and Tn7R extremes, BCD2-*msfgfp* fusion | (4) |
| pBG28 | Km^r^ Gm^r^, ori R6K, pBG derivative vector with 14a synthetic promoter | (4) |
| pBG37 | Km^r^ Gm^r^, ori R6K, pBG-derivative vector with 14c synthetic promoter | (4) |
| pBG42 | Km^r^ Gm^r^, ori R6K, pBG-derivative vector with 14g synthetic promoter | (4) |
| pBG51 | Km^r^ Gm^r^, ori R6K, pBG-derivative vector with 14d synthetic promoter | (4) |
| pGEM-Teasy | Amp^r^, ori ColE1, *lacZa* used for cloning BCD2-*phaZ* fusion | Promega, Wisconsin, USA |
| pMM3 | Amp^r^, pGEM-Teasy derivative vector, BCD2-*phaZ* fusion | This work |
| pMM5 | Km^r^ Gm^r^, ori R6K, pBG-derivative vector with BCD2-*phaZ* fusion | This work |
| pMM8 | Km^r^ Gm^r^, ori R6K, pBG28-derivative vector with BCD2-*phaZ* fusion | This work |
| pMM11 | Km^r^ Gm^r^, ori R6K, pBG37-derivative vector with BCD2-*phaZ* fusion | This work |
| pMM12 | Km^r^ Gm^r^, ori R6K, pBG42-derivative vector with BCD2-*phaZ* fusion | This work |
| pMM14* | Km^r^ Gm^r^, ori R6K, pBG51-derivative vector with BCD2-*phaZ* fusion | This work |

**Table S 1.** Strains and plasmids used in this study. *A point mutation (A🡺G) was observed in the promoter sequence leading to 14d*: TTAATTAATCTACTTGACATCCGACATTCGCGACTGTATAATAAGTTG**G**CCTAGG

**References:**

1. Bagdasarian M, Lurz R, Rückert B, Franklin FC, Bagdasarian MM, Frey J, Timmis KN. 1981. Specific-purpose plasmid cloning vectors. II. Broad host range, high copy number, RSF1010-derived vectors, and a host-vector system for gene cloning in *Pseudomonas*. Gene 16:237–247.

2. Herrero M, De Lorenzo V, Timmis KN. 1990. Transposon vectors containing non-antibiotic resistance selection markers for cloning and stable chromosomal insertion of foreign genes in gram-negative bacteria. Journal of Bacteriology https://doi.org/10.1128/jb.172.11.6557-6567.1990.

3. Boyer HW, Roulland-Dussoix D. 1969. A complementation analysis of the restriction and modification of DNA in *Escherichia coli*. J Mol Biol 41:459–472.

4. Zobel S, Benedetti I, Eisenbach L, De Lorenzo V, Wierckx N, Blank LM. 2015. Tn7-Based Device for Calibrated Heterologous Gene Expression in *Pseudomonas putida*. ACS Synthetic Biology 4:1341–1351.

5. Kessler B, de Lorenzo V, Timmis KN. 1992. A general system to integrate lacZ fusions into the chromosomes of gram-negative eubacteria: regulation of the Pm promoter of the TOL plasmid studied with all controlling elements in monocopy. Mol Gen Genet 233:293–301.

6. Schäfer A, Tauch A, Jäger W, Kalinowski J, Thierbach G, Pühler A. 1994. Small mobilizable multi-purpose cloning vectors derived from the Escherichia coli plasmids pK18 and pK19: selection of defined deletions in the chromosome of *Corynebacterium glutamicum*. Gene https://doi.org/10.1016/0378-1119(94)90324-7.

7. Choi K-H, Gaynor JB, White KG, Lopez C, Bosio CM, Karkhoff-Schweizer RR, Schweizer HP. 2005. A Tn*7*-based broad-range bacterial cloning and expression system. Nat Methods 2:443–448.
