# Supplementary material for: Synthetic Control of Metabolic States in Pseudomonas putida by Tuning Polyhydroxyalkanoate Cycle
Source: mBio. 2022 Jan 18;13(1):e01794-21. doi: 10.1128/mbio.01794-21 (PMC8764540; doi:10.1128/mbio.01794-21)
Supplement: TABLE S3 [file mbio.01794-21-st003.docx]

| Strains | Total Biomass (g/L) | PHA  (% CDW) | PHA  (g/L) | Residual Biomass (g/L) | *(R)*-HAs  (g/L) | Octanoate concentration (mM) |
| --- | --- | --- | --- | --- | --- | --- |
| **t 3h** |  |  |  |  |  |  |
| KT2440 | 0.26 ± 0.02 | 13.25 ± 0.57 | 0.03 ± 0.00 | 0.22 ± 0.01 |  | 13.03 ± 1.21 |
| KT40Z | 0.25 ± 0.02 | 14.48 ± 0.86 | 0.03 ± 0.01 | 0.22 ± 0.02 |  | 14.71 ± 0.59 |
| KT2440 Δ*pha* | 0.26 ± 0.01 | N.D. | N.D. | 0.26 ± 0.01 |  | 15.05 ± 0.99 |
| M1 | 0.26 ± 0.03 | 9.83 ± 0.40 | 0.02 ± 0.00 | 0.23 ± 0.03 |  | 13.52 ± 0.74 |
| M2 | 0.23 ± 0.04 | N.D. | N.D. | 0.23 ± 0.03 |  | 14.63 ± 0.28 |
| M3 | 0.23 ± 0.05 | N.D. | N.D. | 0.23 ± 0.04 |  | 15.39 ± 0.18 |
| M4 | 0.25 ± 0.02 | N.D. | N.D. | 0.25 ± 0.02 |  | 14.80 ± 0.47 |
| **t 5h** |  |  |  |  |  |  |
| KT2440 | 0.52 ± 0.06 | 23.32 ± 2.54 | 0.12 ± 0.01 | 0.40 ± 0.05 | 0.17 ± 0.02 | 11.80 ± 1.44 |
| KT40Z | 0.51 ± 0.03 | 19.09 ± 1.95 | 0.10 ± 0.02 | 0.41 ± 0.02 | 0.00 ± 0.00 | 11.52 ± 1.34 |
| KT2440 Δ*pha* | 0.45 ± 0.01 | N.D. | N.D. | 0.45 ± 0.01 | 0.13 ± 0.01 | 13.64 ± 1.24 |
| M1 | 0.52 ± 0.05 | 20.02 ± 2.16 | 0.11 ± 0.02 | 0.40 ± 0.03 | 0.21 ± 0.02 | 9.85 ± 1.10 |
| M2 | 0.42 ± 0.03 | N.D. | N.D. | 0.42 ± 0.03 | 0.19 ± 0.00 | 12.35 ± 0.84 |
| M3 | 0.40 ± 0.00 | N.D. | N.D. | 0.40 ± 0.00 | 0.16 ± 0.02 | 12.47 ± 0.08 |
| M4 | 0.45 ± 0.06 | N.D. | N.D. | 0.45 ± 0.05 | 0.22 ± 0.01 | 11.88 ± 0.99 |
| **t 10h** |  |  |  |  |  |  |
| KT2440 | 1.22 ± 0.12 | 56.27 ± 2.75 | 0.67 ± 0.04 | 0.52 ± 0.08 | 0.21± 0.02 | 3.13 ± 0.64 |
| KT40Z | 1.08 ± 0.05 | 44.91 ± 3.45 | 0.48 ± 0.03 | 0.59 ± 0.05 | 0.00 ± 0.00 | 3.03 ± 0.54 |
| KT2440 Δ*pha* | 0.61 ± 0.06 | N.D. | N.D. | 0.61 ± 0.05 | 0.14 ± 0.01 | 7.16 ± 0.60 |
| M1 | 1.01 ± 0.08 | 40.26 ± 2.29 | 0.42 ± 0.03 | 0.62 ± 0.03 | 0.37 ± 0.04 | 2.01 ± 0.48 |
| M2 | 0.52 ± 0.01 | N.D. | N.D. | 0.52 ± 0.01 | 0.43 ± 0.04 | 6.32 ± 0.30 |
| M3 | 0.51 ± 0.05 | N.D. | N.D. | 0.51 ± 0.04 | 0.48 ± 0.02 | 5.43 ± 0.35 |
| M4 | 0.56 ± 0.05 | N.D. | N.D. | 0.56 ± 0.04 | 0.48 ± 0.04 | 6.55 ± 0.43 |

**Table S 3. Physiological data at different time points during the growth curve.** They were used in the in-silico contextualization process under PHA accumulation condition. N.D.: not detected
